# The Landscape of Immune Cells Infiltrating in Prostate Cancer

**DOI:** 10.3389/fonc.2020.517637

**Published:** 2020-10-29

**Authors:** Zhicong Wu, Hua Chen, Wenyang Luo, Hanyun Zhang, Guihuan Li, Fangyin Zeng, Fan Deng

**Affiliations:** ^1^ Department of Clinical Laboratory, Nanfang Hospital, Southern Medical University, Guangzhou, China; ^2^ Department of Clinical Laboratory, The Fifth Affiliated Hospital, Southern Medical University, Guangzhou, China; ^3^ Department of Cell Biology, School of Basic Medical Sciences, Southern Medical University, Guangzhou, China

**Keywords:** infiltration, immune cell, prostate cancer, CIBERSORT, nomogram

## Abstract

**Background:**

This study was to explore the infiltration pattern of immune cells in the prostate cancer (PCa) microenvironment and evaluate the possibility of specific infiltrating immune cells as potential prognostic biomarkers in PCa.

**Methods:**

Infiltrating percentage of 22 immune cells were extracted from 27 normalized datasets by CIBERSORT algorithm. Samples with CIBERSORT *p*-value < 0.05 were subsequently merged and divided into normal or tumor groups. The differences of 22 immune cells between normal and tumor tissues were analyzed along with potential infiltrating correlations among 22 immune cells and Gleason grades. SNV data from TCGA was used to calculate the TMB score. A univariate and multivariate regression were used to evaluate the prognostic effects of immune cells in PCa.

**Results:**

Ten immune cells with significant differences were identified, including seven increased and three decreased infiltrating immune cells from 190 normal prostate tissues and 537 PCa tissues. Among them, the percentage of infiltration of resting NK cells increased the most, whereas the percentage of infiltration of resting mast cells decreased the most. In normal tissues, CD8+ T cells had the strongest infiltrating correlation with monocytes, while activated NK cells and naive B cells were the highest in PCa tissues. Moreover, the infiltration of five immune cells was significantly associated with TMB score and mutations of immune gene change the infiltration of immune cells. The Area Under Curve (AUC) of the multivariate regression model for the five- and 10-year survival prediction of PCa reached 0.796 and 0.862. The validation cohort proved that the model was reproducible.

**Conclusions:**

This study demonstrated that different infiltrating immune cells in prostate cancer, especially higher infiltrating M1 macrophages and neutrophils in PCa tissue, are associated with patients’ prognosis, suggesting that these two immune cells might be potential targets for PCa diagnosis and prognosis of treatment.

## Introduction

As a common cancer in males, prostate cancer (PCa) is highly diagnosed in both the United States and Europe, accounting for 10% of cancer-related deaths in males (1, 2). Although the incidence of PCa in Asia is much lower than that in the United States and Europe, the incidence and mortality of PCa is rising steadily in China (3). At present, there are few efficient clinical treatments for PCa, most of which are radical prostatectomy and androgen deprivation therapy (ADT). But the treatment effect is not satisfactory (4, 5). Therefore, new therapeutic targets and prognostic markers are needed for PCa therapy.

Numerous studies have now documented that tumor-infiltrating immune cells (TIICs) have a link with prognosis and response to immunotherapy in several human carcinoma types. In PCa, TIICs also play an essential role in PCa progression, which include T cell, B cell, mast cell, NK cell, etc. TIICs have different functions and compositions in different stages of PCa. In PCa tissues, CD4+ T cells are involved in PCa progression, and some data showed that the infiltration of CD4+ T cells is increased in PCa tissues and promotes PCa metastasis (6). Invariant Natural Killer T (iNKT) cells were found to delay PCa progression by sculpting the TME (7). In the procession of PCa metastasis patients, mast cells could enhance PCa resistance to chemotherapy and radiotherapy, and eventually progress to metastasis stage (8). The infiltration of CD8+ tumor-infiltrating lymphocytes (TILs) in invasive margins of PCa may be related to the poor prognosis of PCa patients (9). Although there are many known relations between immune cells (10, 11), the immune system contains various types of immune cells, which constitute a complex regulatory network, and so far many modulatory synergies between immune cells remain unclear due to a complex regulatory network in tumor immune microenvironment. Therefore, it is necessary to conduct an in-depth analysis of major immune cells and their subtypes infiltrating in PCa tissues, and to analyze potential internal relationships. In addition, there are few studies about infiltrating immune cells in PCa, and there is also no effective method for analyzing immune cells infiltration in the microenvironment of cancer tissues in an efficient, rapid way.

CIBERSORT is a tool for deconvolving the infiltration matrix of 22 immune cells, which is based on the principle of linear support vector regression, only using RNA-Sequence data to estimate immune cells infiltration by identifying more than 500 marker genes (12). The tool was first published in 2015 and is the most-cited tool for analyzing immune cells estimation infiltration so far.

In this study, we systematically analyzed the proportion of 22 immune cells infiltrating in the normal prostate and PCa microenvironment by CIBERSORT algorithm, and then explored the correlation of infiltrating immune cells with malignancy, survival time, immunotherapy, and genomic mutation. In addition, we evaluated 22 immune cells serving as potential prognostic biomarkers in prostate cancer. This study will help to improve our understanding of immune cells in the PCa microenvironment and provide potential new therapeutic targets for PCa.

## Material and Methods

### Gene Expression Datasets and Normalization

The data for this study was originated from public databases. Gene expression profile data were downloaded from TCGA (https://portal.gdc.cancer.gov/) and GEO (https://www.ncbi.nlm.nih.gov/geo/). This study focused on the difference in immune cell infiltration between primary prostate cancer tissue and normal prostate tissue. Datasets included in this study were based on the following criteria: (1) The organism of samples is Homo sapiens. (2) The source of the samples must be normal prostate tissue or prostate cancer tissue. (3) The dataset contains more than 6 samples. (4) Samples were not treated with medication, radiation, and chemotherapy before sampling. (5) Samples are from the USA and European countries. All samples’ genes were represented by gene symbol. If a gene matched multiple probes, the median value of multiple probes expression was applied. Then, all datasets were normalized by the limma package of the R language. In total, 5,423 samples from 27 datasets were included in this study; details of datasets are shown in **Table 1**. All samples’ ID and samples applied for each analysis are shown in **Supplementary Table 1**.

**Table 1:** 27 datasets used for deconvolution of immune cells profiles.

Group	Datasets
Normal	TCGA, GSE26910 (13), GSE32448 (14), GSE2443 (15), GSE5132 (16), GSE71016 (17),GSE15484 (18), GSE32571 (19), GSE38241 (20), GSE41969 (21), GSE62872 (22),GSE70768 (23), GSE29079 (24), GSE7220 (25), GSE77959 (26)
Tumor	TCGA, GSE41969 (21), GSE44353 (27), GSE6606 (28), GSE6287 (22), GSE64333 (29),GSE7055 (30),GSE68555 (31),GSE68882 (32), GSE70768 (23), GSE71783 (29), GSE1431 (33), GSE16120 (34),GSE15484 (18), GSE16560 (35), GSE2443 (15), GSE26242, GSE26910 (13), GSE32448 (14), GSE32571 (19),GSE35988 (36)

### Inference of 22 Immune Cells Infiltrating Proportion

Normalized gene expression profile data were used to obtain 22 immune cells infiltration proportion by CIBERSORT package in R. The CIBERSORT algorithm based on deconvolution principle was used to quantify 22 immune cells infiltrating proportions by RNA expression profiles. The CIBERSORT algorithm implements support vector regression by identifying 547 immune cell-related genes, which improved the performance and accuracy of deconvolution. Samples with CIBERSORT *p*-value < 0.05 were considered eligible for subsequent analysis. To ensure the results from CIBERSORT more accurately, we used the “relative mode” of CIBERSORT, which is the relative proportion of 22 immune cells in human tissue that can be analyzed, and the sum of the infiltration percentage of all immune cells is 100%. Twenty-two immune cells include naive B cells, memory B cells, plasma cells, CD8+ T cells, naive CD4+ T cells, resting CD4+ memory T cells, activated CD4+ memory T cells, follicular helper T cells, regulatory T cells (Tregs), gamma delta T cells, resting Natural Killer (NK) cells, activated NK cells, monocytes, M0 macrophages, M1 macrophages, M2 macrophages, resting dendritic cells, activated dendritic cells, resting mast cells, activated mast cells, eosinophils, and neutrophils, which are the majority of innate immune and adaptive immune cells in the human body. In total, 190 normal samples and 537 cancer samples with CIBERSORT *p*-value<0.05 were included.

### Infiltrating Differential Analysis of 22 Immune Cells

The samples with CIBERSORT *p*<0.05 from different datasets were combined and divided into normal and cancer groups.

The histogram was drawn by R software to describe the overall distribution of 22 immune cells. Differential analysis was performed in the two groups using an independent *t*-test. The Vioplot package was then used for visual plotting. To observe which combination of immune cells simultaneously up-regulated or down-regulated, the Corrplot package was used to examine the correlation coefficient between 22 immune cells in two groups, respectively.

### Analysis of the Correlation Between Immune Cells and PCa Malignancy Grade

Gleason grade score was used to describe the malignancy of PCa. The 203 samples from GSE44353(91), GSE7055(39), GSE68555(42), and GSE70768(31) datasets with CIBERSORT *p*-value<0.05 and Gleason grade score were included. Gleason grade scores were divided into four groups of ranking 6 to 9. Details of samples were shown in **Supplementary Table 1**.

### Correlation Between Infiltrating Immune Cells and Tumor Mutational Burden

Tumor mutational burden (TMB) is defined as the number of somatic, coding, base substitution, and indel mutations per megabase of genome examined. Single nucleotide variants (SNV) data for TMB analysis were downloaded from TCGA (https://portal.gdc.cancer.gov/). TMB score was calculated by the formula: the observed number of mutations divide 38Mb (the size of the exome capture)=SNV/MB ratio (37). Then, 42 samples from TCGA with CIBERSORT *p*-value<0.05 together with effective TMB score were included. Correlation of TMB and 22 immune cells was displayed by the Pearson correlation coefficient. GraphPad prism (8.0.2) and the maftools package in R was used for plotting.

### Key Immune Genes Screening and Mutation Analysis

The list of 2,498 immune genes was downloaded from the IMMPORT database (38). The immune gene expression profile was extracted from TCGA RNA data. Then, the limma package was used for differential immune gene analysis between normal tissues and PCa tissues. Immune genes with the absolute value of log_2_foldchange more than 1 and adjusted *p*-value<0.05 were identified as differential immune genes. Further, the survival package was used for screening for immune genes related to survival. The differential immune genes with univariate Cox regression *p*-value<0.05 were eventually identified as key immune genes. Subsequently, the TIMER website (39) was used to analyze the correlations of key immune genes mutation and the infiltration of immune cells. The mutation type was automatically recognized by the website. The heatmap was plotted by the pheatmap package and the volcano figure was plotted by the ggplot2 package.

### Survival Analysis and Survival Prediction of PCa Patients

The samples with CIBERSORT *p*-value<0.05 together with available follow-up data in cancer group was used for survival analysis. Forty-four samples from TCGA and 34 samples from GSE16560 were applied for survival analysis and prediction. Overall survival (OS) time was used to monitor the life quality of patients with different infiltrating immune cells. Seventy-eight samples were randomly divided into training group and validation group by SPSS software. A multivariate regression model was constructed to calculate the risk score of training group. Risk score = β_1_*X_1_ + β_2_*X_2_ + β_3_*X_3_ +… β_i_*X_i_, with β representing the coefficient of multivariate regression and X representing the expression of factors included in the multivariate regression equation. The samples in the training cohort were divided into high risk and low risk groups by the median of the risk score, and a Kaplan-Meier curve was then plotted to evaluate survival differences. Then, a nomogram based on the multivariate regression model was built to predict five- and ten-year survival probability by immune cells infiltration percentage and patient age. Then, the Calibration graph and Receiver Operating Characteristic (ROC) curve were used to evaluate the prediction model. Subsequently, the risk score of the validation group was calculated by the equation using the coefficient from the training group. Likewise, the Kaplan-Meier curve and ROC curve were used to evaluate the prediction effectiveness of the validation group.

### Statistical Analysis

Statistical analyses were performed on R(3.6.1) and SPSS21.0. Two-tailed independent *t*-test was used to test the differences between normal and tumor groups, with Bonferroni adjustment used for multiple comparisons. Comparison between multiple groups used the Kruskal-Wallis test. Correlations were assessed by the Pearson correlation coefficient. Cohen’s kappa test was used to test coincidence. Survival analysis between normal and tumor groups was conducted by log-rank test. For multiple comparisons, Bonferroni adjusted *p*-value<0.05/(number of tests) was considered statistically significant. For other tests, *p*-value<0.05 was considered statistically significant.

## Results

### Different Infiltration Proportion of Immune Cells Between PCa and Normal Tissues

**Figure 1:**
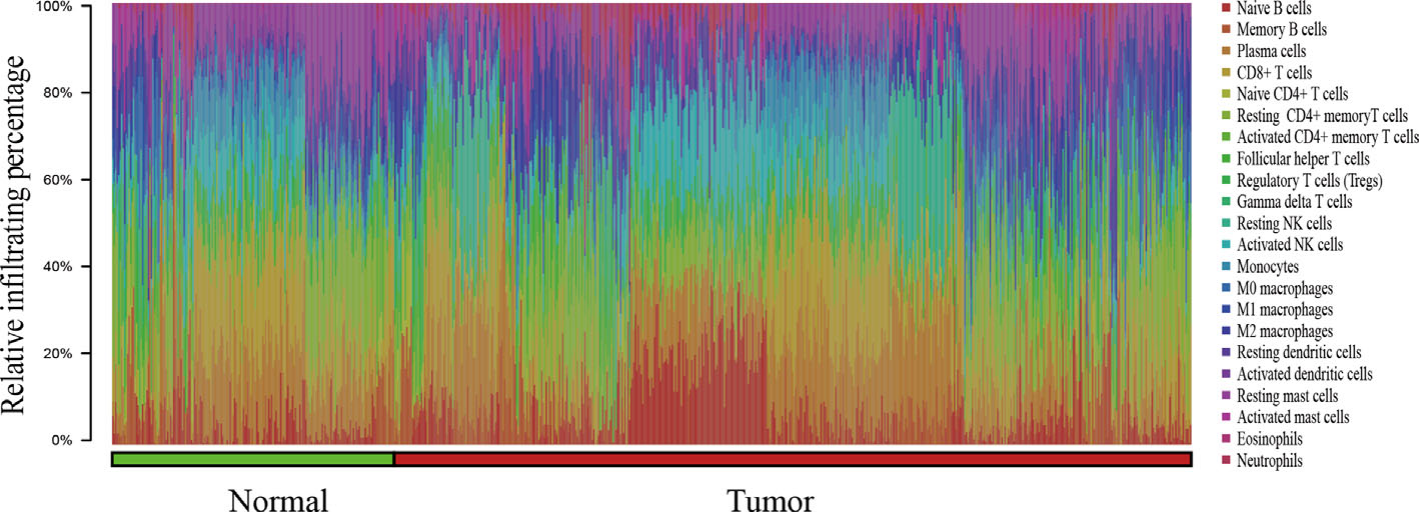
The bar chart summarizes the percentage of 22 infiltrated immune cells from normal (n=190) and PCa (n=537) tissues. Each color represents a kind of immune cell, and the length of the bar represents the relative percentage of infiltrating immune cells. The proportion of each infiltrated immune cell is expressed as a percentage of the total 22 immune cells, and the sum infiltrating percentage of 22 immune cells is 100%.

**Figure 2:**
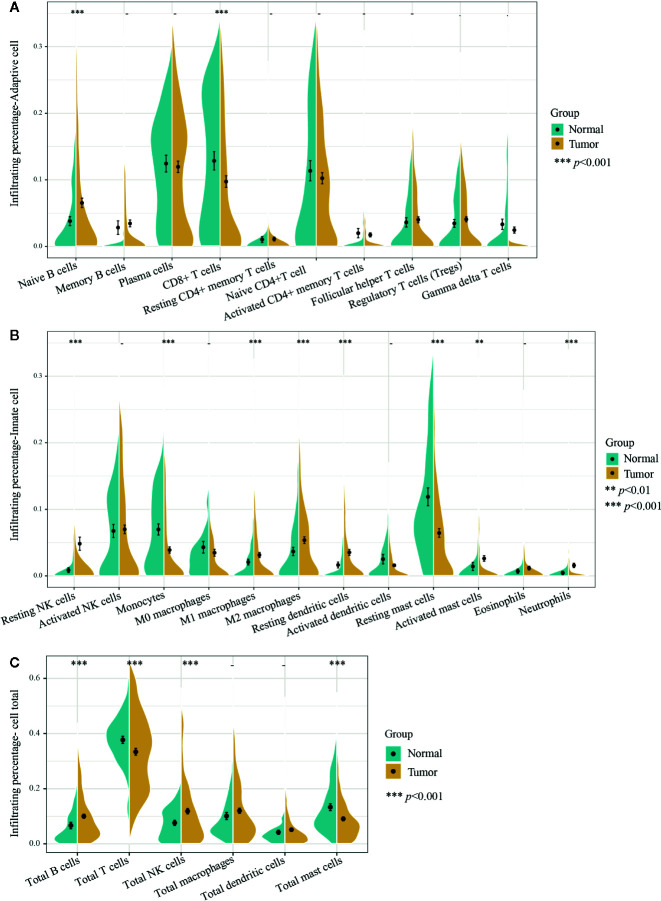
Infiltrating differences of 22 immune cells and six kinds of total immune cells between normal and PCa tissues. **(A)** Ten adaptive immune cells and **(B)** 12 innate immune cells infiltration percentages were calculated by the CIBERSORT algorithm. The infiltrating percentages of the **(C)** six total immune cells were obtained by adding all their subtypes from 22 immune cells. Yellow represents PCa tissues (n=537) and blue represents normal prostate tissues (n=190). Two-tailed independent *t*-test was applied. *p*-values of differences between groups are shown above the violin plot. After Bonferroni adjusted, immune cells (left side) with *p*-value < 0.00227 were considered statistically significant, immune cells total (right side) with *p*-value < 0.00833 were considered statistically significant. **p < 0.01, ***p < 0.001.

**Figure 3:**
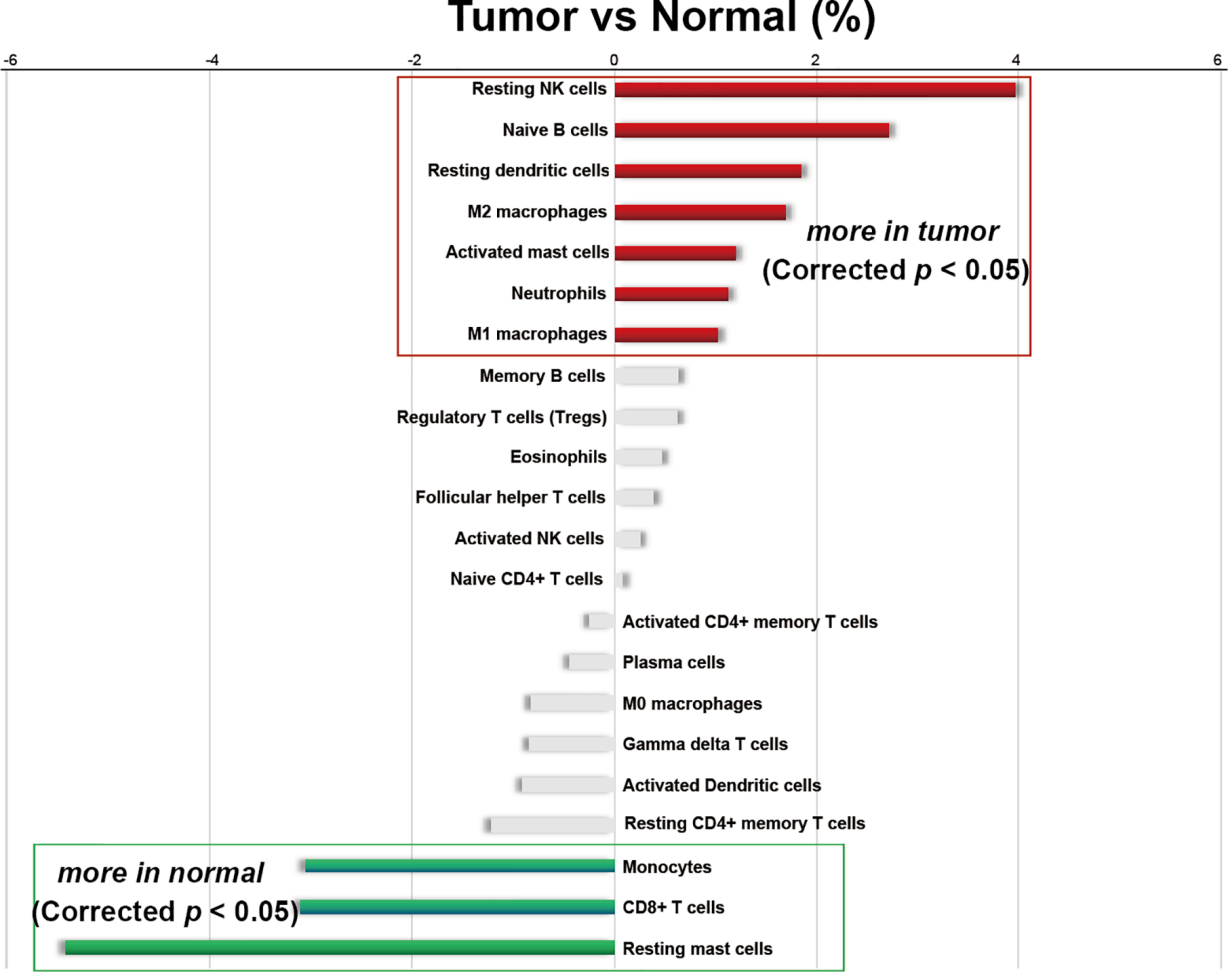
Differences in the average infiltrating proportion of immune cells in normal and PCa tissues. The calculation formula is the mean value of the percentage infiltration in the tumor group (n=537) minus the mean value of the percentage infiltration in the normal group (n=190). All *p*-values were corrected by Bonferroni adjustment. The left panel of the coordinate axis represents the higher for infiltrating percentage of immune cells in normal tissues than that in cancer tissues, and the right panel is higher for infiltrating percentage of immune cells in cancer tissue than that in normal tissues. Red and green bars represent immune cell types with significantly up-regulated and down-regulated between two groups, respectively.

In this study, a total of 727 effective samples were obtained through the CIBERSORT algorithm, including 190 normal prostate tissues and 537 PCa tissues (**Figure 1**). As shown in **Figure 2** and **Supplementary Table 2**, the infiltration proportion of CD8+ T cells (12.8%) was highest, and that of neutrophils (0.5%) was the lowest in normal prostate tissues. By contrast, the infiltration proportion of plasma cells (12.0%) was the highest while resting CD4+ memory T cells (0.2%) were the lowest in PCa. After Bonferroni adjustment, the difference in the mean infiltrating proportion of the same immune cell in two groups was shown in **Figure 3**.

Moreover, among the 22 immune cells, the infiltration proportion of seven kinds of immune cells in PCa tissues including resting NK cells, naive B cells, resting dendritic cells, M2 macrophages, activated mast cells, neutrophils, and M1 macrophages were significantly higher than that in normal tissues. In contrast, the infiltration proportion of monocytes, CD8+ T cells, and resting mast cells in normal tissues were remarkably elevated compared with that in PCa. There was no difference in the infiltration proportion of the remaining 12 immune cells between two groups (**Figure 2**, **Figure 3**, and **Supplementary Table 2**). These results demonstrated that there were different infiltration patterns of immune cells infiltrating in normal prostate tissue and PCa tissue.

### Difference of Adaptive Immune Cells in PCa and Normal Tissues

Adaptive immune cells include plasma cells, T cells, and B cells and its subtypes. We first compared the different infiltrating proportions of adaptive immune cells between normal and PCa tissues. As shown in **Figures 2A, C**, the infiltrating percentage of total T cells in PCa tissues was significantly lower while total B cells were remarkably higher than that in normal tissues, but there was no difference in the proportion of plasma cells.

Further analysis of the subtypes of B cells showed that the increased infiltration proportion of total B cells in PCa tissues was due to the immature type of B cells (naive B cells), which was 2.7% higher than that in normal tissue, and the memory B cells were basically consistent between groups. Interestingly, the decline of total T cells was mainly due to decreased infiltration of CD8+ T cells, which decreased by 3.1% in PCa tissues. Other subtypes of T cells did not differ significantly between the two groups. Notably, T cells were the only immune cell with the highest infiltrating proportion in both normal and cancer tissues. Especially in normal tissues, the infiltrating percentage of total T cells accounted for nearly 40% of the total infiltration proportion of 22 immune cells.

### Difference of Innate Immune Cells in Normal and PCa Tissues

We then analyzed the difference of innate immune cells including macrophages, monocytes, mast cells, NK cells, neutrophils, eosinophils, and dendritic cells along with its subtypes in normal and PCa tissues. As also shown in **Figure 2B**, there was a different infiltrating rate in the different subtypes of some innate immune cells. For example, both M1 and M2 macrophages were significantly up-regulated in PCa tissues. However, there was no statistical difference in the infiltration rate of M0 macrophages and the total macrophages. Moreover, the infiltrating proportion of total NK cells and resting dendritic cells were significantly increased over that in normal tissue, and the infiltrating rate of activated mast cells was significantly increased over that in normal tissues. Of note, the proportion of total NK cells was significantly higher in PCa tissues (**Figure 2C**), and the resting NK cells contributed the major increment, with nearly 4% of total immune cells percentage, the largest increased infiltration proportion in cancer tissues.

### Infiltration Correlation of 22 Immune Cells

To evaluate whether 22 kinds of immune cells showed convergence during infiltration, correlation analysis was used to speculate the potential one-to-one association. The absolute value of the correlation coefficient between 0.10 and 0.39 represents a weak correlation, 0.40 to 0.69 represents a medium correlation, and 0.7 to 0.89 represents a strong correlation (40).

**Figure 4:**
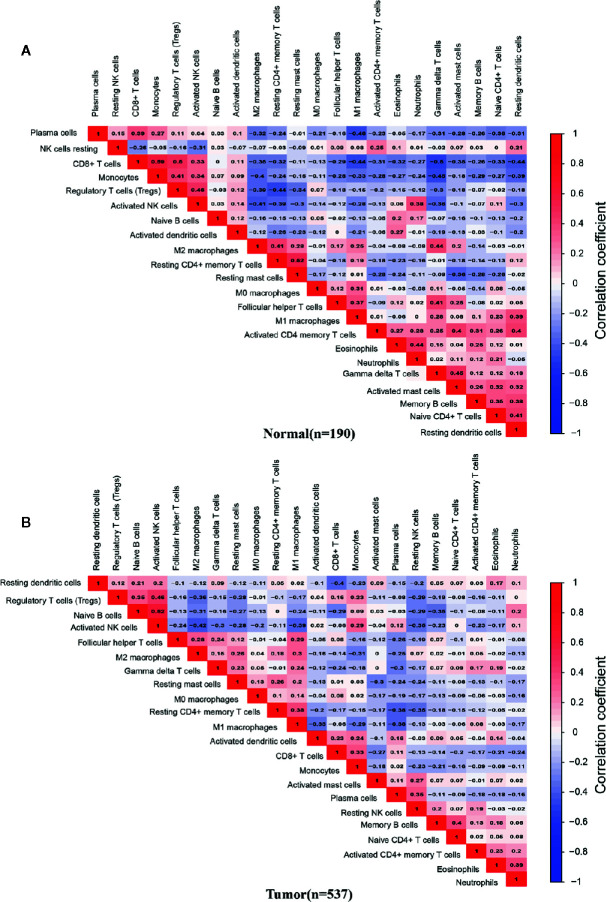
Correlation matrix of 22 infiltrating immune cells in normal and PCa tissues. The numbers in the two matrices represent the Pearson correlation coefficient. Red box represents a positive correlation, blue box represents a negative correlation, and white box represent no correlation between two kinds of cells. **(A)** Normal prostate tissues (n=190). **(B)** Prostate cancer tissues (n=537).

We analyzed the correlation between the infiltrating percentage of 22 immune cells in normal tissues (n=190) and PCa tissues (n=537), respectively. In normal tissues, the positive correlation between CD8+ T cells and monocytes was the strongest, in which correlation coefficient was 0.59. And the correlation coefficient between gamma delta T cells and CD8+ T cells was -0.5, the lowest negative correlation (**Figure 4A**). However, in PCa tissues, the positive correlation between activated NK cells and naive B cells was the strongest, more than 0.6, and the cells with the strongest negative correlation were activated NK cells and M2 macrophages (**Figure 4B**).

### Association of Infiltrating Immune Cells and PCa Malignancy Grades

**Figure 5:**
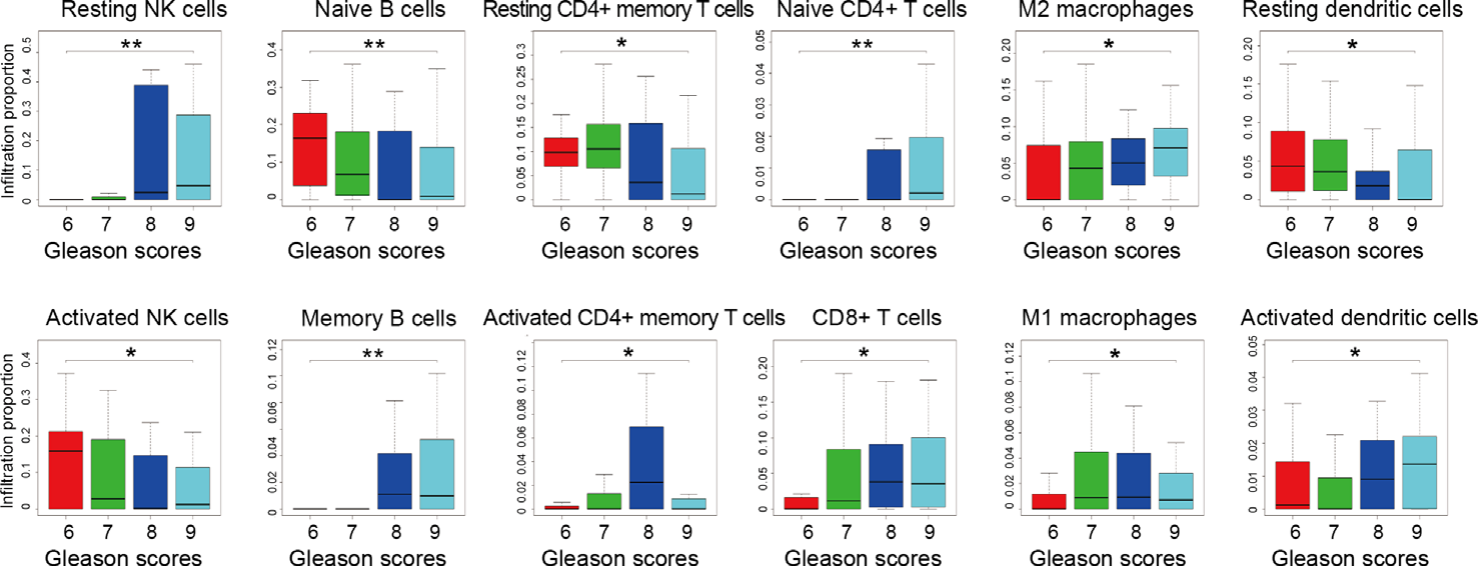
Infiltration proportion of different immune cells from prostate cancer patients with different Gleason grades. Four datasets of GSE44353 (n=91), GSE7055 (n=39), GSE68555 (n=42), and GSE70768 (n=31) were included. The immune cell infiltration percentage was calculated by the CIBERSORT algorithm. The number of patients with 6, 7, 8, 9 Gleason grade score is 55, 113, 15, 20, respectively. Kruskal-Wallis test was applied. *p < 0.05, **p < 0.01.

Gleason grade is an essential index in the diagnosis and treatment of PCa, the higher the malignancy of prostate cancer, the higher the Gleason grade score (41). To further explore the association between infiltrating immune cells and PCa malignancy grades, we extracted 203 samples’ infiltration matrix data from PCa patients with Gleason grade data. Twelve types of immune cells in PCa showed significant differences in the four Gleason grades (**Figure 5**). It is worth noting that resting NK cells was not only the most significant difference in infiltration percentage between the two groups but also the most obvious Gleason gradation and differentiation in PCa.

Meanwhile, by analyzing the distribution map of Gleason graded immune cells, the changes of each immune cell in different Gleason grades can be preliminarily understood. The infiltration trend of resting NK cells, memory B cells, M2 macrophages, CD8+ T cells, and activated dendritic cells were positively correlated with the malignant degree of PCa. However, naive B cells, activated NK cells, and resting dendritic cells were negatively correlated with the degree of malignancy. These results suggest that there is more than one type of immune cell associated with PCa malignancy, and there may be various types of immune cells and components involved in PCa grading.

### The Effect of Tumor Mutational Burden (TMB) and Immune Genes Mutations on the Infiltration of Immune Cells

Given that all immune cells are not associated with PCa malignancy based on the above data, a lack of tumor neoantigen is associated with reduced immune cell infiltration in the lung cancer microenvironment (42). However, it is not clear which types of immune cells are affected by TMB in the PCa microenvironment. Here, we aimed to identify which infiltration of immune cells was affected by TMB in PCa.

**Figure 6:**
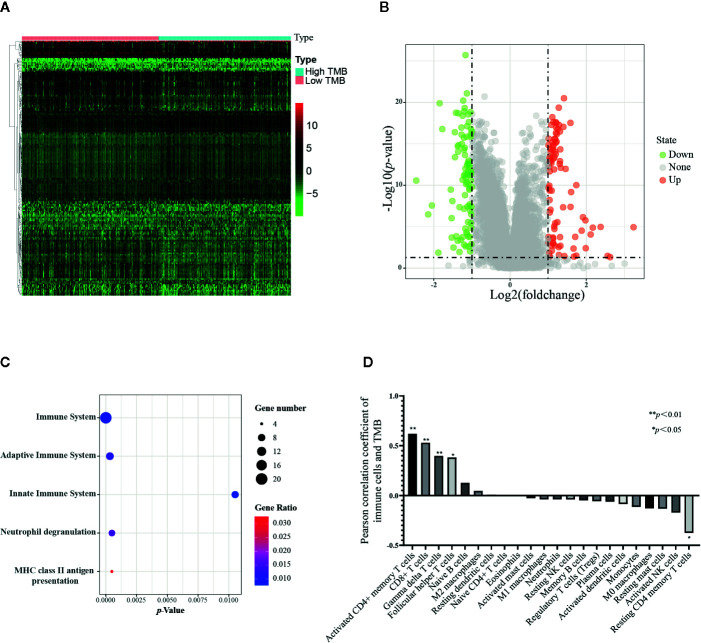
Correlation of TMB with 22 infiltrating immune cells in prostate cancer. **(A)** Heatmap of 178 differential genes in low TMB samples(n=247) and high TMB samples (n=239) from TCGA RNA expression data. **(B)** Volcano plot of differential genes. **(C)** Bubble map of immune-related functional enrichment analysis. The immune genes with absolute value of log_2_ fold change more than one and adjusted *p* value less than 0.05 were included for functional enrichment analysis. **(D)** Correlation analysis between TMB and immune cells, 42 tumor samples from TCGA were applied. The Pearson correlation coefficient greater than 0 represents a positive correlation between TMB and 22 immune cells, and the correlation coefficient less than 0 represents a negative correlation. *p < 0.05, **p < 0.01.

The TCGA samples with available RNA expression data were divided into high TMB and low TMB groups according to the median of TMB (**Figure 6A**), and 178 genes were significantly different in these two groups (**Figure 6B**). Then, functional enrichment analysis was performed on the KOBAS 3.0 online database (43), and the immune-related results demonstrated that these 178 differential genes were involved in both adaptive immune system and innate immune system (**Figure 6C**). We further combined the TMB data and immune cells infiltration data from the same TCGA patient. Forty-two samples together with TMB data and CIBERSORT *p*-value<0.05 were included for the following analysis. The TMB score of 42 samples ranged from 0.02 to 236, the types and classifications of SNV type and classification were shown in **Supplementary Figure 1**. Missense mutations occurred most frequently in PCa, and the mutation frequency of the TP53 gene was the highest. As shown in **Figure 6D**, there was a strong correlation between infiltrating T cells and TMB scores. With the increase of TMB, the infiltration of four subtypes of T cells, including activated CD4+ memory T cells, CD8+ T cell, gamma delta T cells, and follicular helper T cells were increased, while only the infiltration of resting CD4+ memory T cell decreased with the increase of TMB score. These results suggest that TMB may be closely related to T cell infiltration. Given the low infiltration of these five cells in PCa tissues, these immune cells may be potential targets for immunotherapy for PCa.

**Figure 7:**
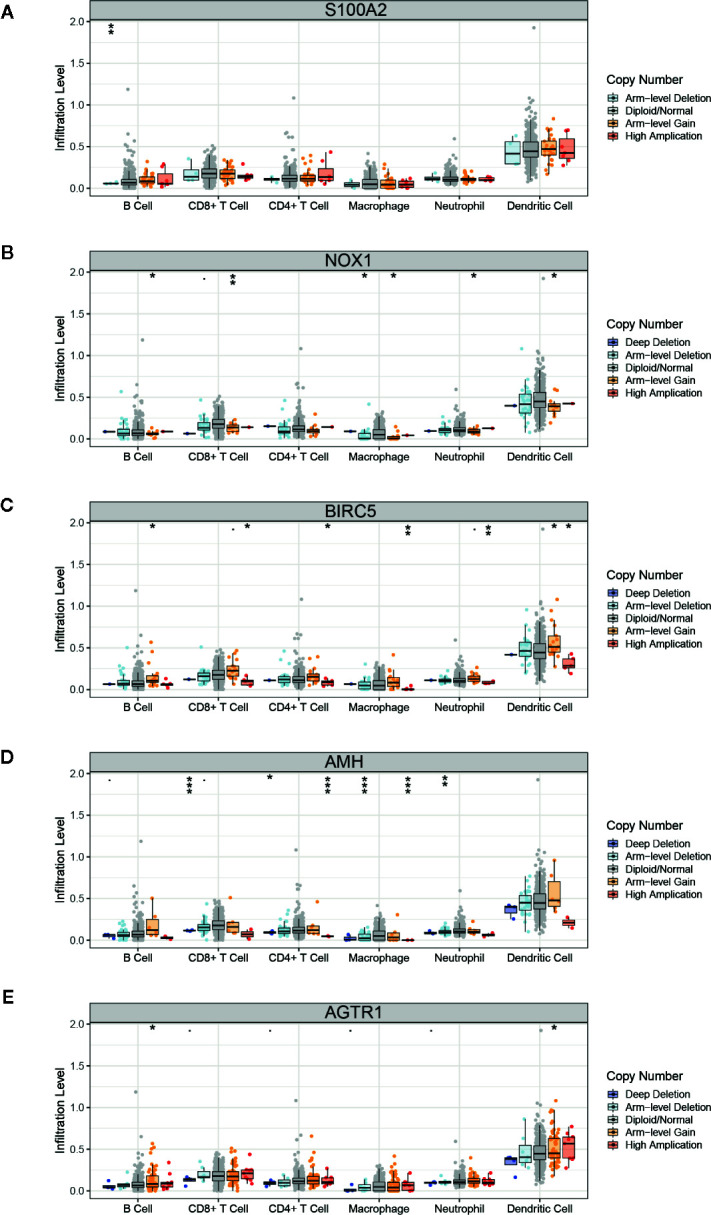
Correlation of immune gene mutations with infiltrating immune cells. **(A–E)** Mutation of five key immune genes corresponding infiltration level. Box plots are presented to show the distributions of each immune subset at each copy number status in prostate cancer. The infiltration level for each mutation category is compared with the normal using a two-sided Wilcoxon rank-sum test. *p < 0.05, **p < 0.01, ***p < 0.001.

The TMB score was associated with the mutation of overall genes, and we hypothesized the mutation of single immune gene may also relate to immune cells infiltration. The list of 2,498 immune genes was downloaded from the IMMPORT database. Then we conducted a differential analysis of the immune genes’ expression in normal tissues and PCa tissues (**Supplementary Figure 2A**), and 193 differential immune genes were identified (**Supplementary Figure 2B**, **Supplementary Table 3**). Subsequently, we applied a univariate Cox regression to screen the key immune genes that have an impact on patient survival (**Supplementary Figure 2C**). The SCNA module of TIMER database was used to evaluate the impact of the 5 key immune genes mutation on the infiltration of immune cells. As shown in **Figure 7**, compared with the infiltrating level in samples with wild type of immune genes, mutation carried by genes of S100A2, NOX1, and AMH could commonly inhibit the immune infiltrates. However, the mutations of BIRC5 and AGTR1 increased the infiltration of the immune cells. The dual effect of single gene mutation revealed that the mutation affected the normal immunological function of the genes and reduced the infiltration of immune cells, but neoantigen generated by mutation may serve as new targets and increased immune cell infiltration. Furthermore, the same mutation occurred in the same immune gene caused the different immune cell infiltration to change simultaneously. Therefore, mutations of immune genes may be one of the mechanisms by which different immune cells have an infiltration correlation.

### Association of Infiltrating Immune Cells and PCa Patients’ Survival

**Figure 8:**
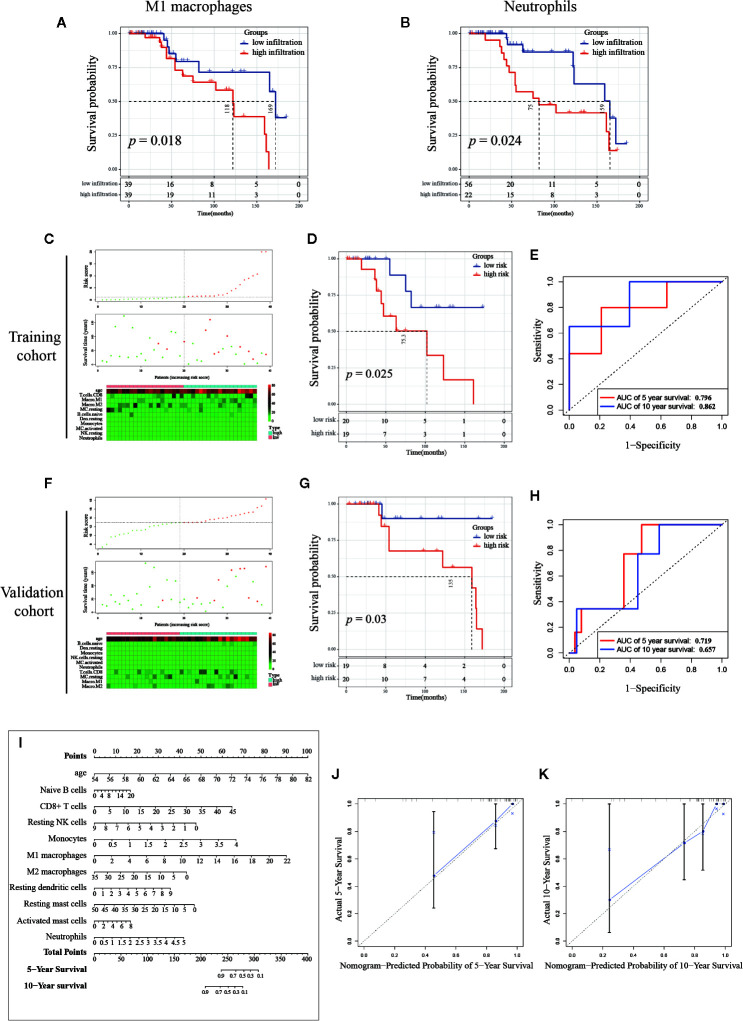
Establishment and validation of the prognosis signature based on infiltrating immune cells. **(A, B)** Kaplan-Meier curve of M1 macrophages and neutrophils, PCa samples (n=78) were seriated by the percentage of infiltration per immune cell, with the top half defined as the high-infiltration group and the last half defined as the low-infiltration group. (Note: the infiltration percentage of neutrophils in 56 samples were 0%, and these samples were categorized into low-infiltration group. Details of the number of samples were shown in the figure.) **(C, F)** The distribution of sample with high and low risk score calculated by multivariant Cox model. **(D, G)** The Kaplan-Meier curve of between high and low risk group, and the **(E, H)** Receiver Operating Characteristic curve of five-year and 10-year predicted prognosis in the training cohort and validation cohort, respectively. **(I)** Combinations of 10 immune cells and age were used to predict 5-year and 10-year survival outcomes of PCa patients by nomogram based on multivariate Cox regression model. The unit of immune cells was the infiltrating percentage. The infiltrating percentage of immune cells and age can get a point corresponding to the uppermost scoring axis. Points of the 13 indicators were added to obtain the total points, and the value corresponding to the total points was the five-year or 10-year survival probability. **(J, K)** Calibration graph of five-year and 10-year predicted prognosis.

As mentioned above, immune cells infiltrating into PCa tissue have a certain effect on cancer progression, and we then explored the effectiveness of single or multiple immune cells in the PCa diagnosis, and the infiltrating percentage of multiple immune cells as a potential prognostic prediction index of PCa. First, we investigated the effect of a single type of immune cell infiltration proportion on patients’ survival. The Kaplan–Meier (K-M) survival analysis was separately utilized to analyze the outcome of 10 immune cells with differential infiltration. As shown in **Figures 8A, B**, the high infiltration of M1 macrophages and neutrophils significantly reduced the survival time of PCa patients, suggesting that these two immune cells are potential therapeutic targets for PCa.

### Establishment and Validation of Survival Prediction Model Based on Infiltrating Immune Cells

Mounting evidence showed that age is a risk factor for PCa (44–46). To exclude multi-collinearity, the multivariate regression model included 10 immune cells with inter-group differences. Subsequently, we collected the samples with available survival data and age information. In total, 78 samples were included and then randomly divided into training cohort and validation cohort by SPSS software (seed: 99). A multivariate regression model was constructed to calculate the risk score of samples in the training cohort using the survival package. Risk score = 0.1574*(age) + 0.0372*(Naive B cells) + 0.0632*(CD8+ T cells) - 0.2332*(Resting NK cells) + 0.7313*(Monocytes) + 0.1803*(M1 macrophages) - 0.0545*(M2 macrophages) + 0.1744*(Resting dendritic cells) - 0.0413*(Resting mast cells) + 0.0936*(Activated mast cells) + 0.3688*(Neutrophils), details are shown in **Table 2**. In the training cohort, samples were divided into high risk and low risk groups according to risk scores (**Figure 8C**). Survival analysis showed that the group with a high risk score had a worse prognosis (**Figure 8D**), and time-dependent ROC curve demonstrated that the AUC of 5-year and 10-year training survival model reached to 0.796 and 0.862 respectively (**Figure 8E**), indicating that the multivariate regression model had a high diagnostic efficiency. Next, the equation was applied to calculate the risk score of samples in the validation cohort. Same as the training cohort, samples in the validation cohort were divided into two groups (**Figure 8F**). Also, the two groups showed different survival trends (**Figure 8G**) though the prediction efficiency was lower than the training cohort (**Figure 8H**).

**Table 2:** Details of the multivariate regression model.

Factor	Coefficient	HR	HR.95L	HR.95H	*p*-value
Age	0.1574	1.1704	0.9910	1.3823	0.0638
Naive B cells	0.0372	1.0379	0.7218	1.4922	0.8410
CD8+ T cells	0.0632	1.0652	0.9110	1.2456	0.4284
Resting NK cells	-0.2332	0.7920	0.3199	1.9608	0.6141
Monocytes	0.7313	2.0778	0.8000	5.3966	0.1332
M1 macrophages	0.1803	1.1975	0.9353	1.5334	0.1529
M2 macrophages	-0.0545	0.9470	0.8248	1.0874	0.4400
Resting dendritic cells	0.1744	1.1905	0.7533	1.8815	0.4551
Resting mast cells	-0.0413	0.9595	0.7314	1.2589	0.7656
Activated mast cells	0.0936	1.0981	0.5675	2.1249	0.7810
Neutrophils	0.3688	1.4460	0.3643	5.7400	0.6000

To visualize the multivariate regression model more intuitively, we plotted the nomogram to display the model (**Figure 8I**). The calibration diagram was used to internally verify the model fitting effect. As shown in **Figures 8J, K**, the predicted survival probability from the model was fit well to the actual survival probability, which proved that this model was effective and indicated that it had potential value in clinical promotion. It is worth noting that the cohort we use here contains only 78 samples. If there are more samples containing survival data, the nomogram will be further optimized and the analysis of survival prognosis prediction will be more accurate. Our results suggest that the identification and combined analysis of multiple immune cells are helpful for the classification and prognosis prediction of PCa.

## Discussion

Immunotherapy has become one of the hottest fields of cancer research in the last decade. Since 2011, various immune checkpoint (ICB) inhibitors such as PD1 and CTLA-4 monoclonal antibody and adaptive immune cell therapy such as CAR-T, CAR-NK, and Lymphokine-Activated Killer cells (LAK) have achieved a good clinical therapeutic effect in the treatment of malignant tumors like melanoma, lung cancer, gastric cancer, and breast cancer. However, for patients with PCa, the therapeutic effects of the above two methods are limited (47). PCa is defined as “cold tumor” based on its immunological and genomic characteristics (48), which means the infiltrating immune cells in the tumor microenvironment are lower and fail to initiate, resisting immunotherapy. Therefore, in-depth study of immune cells such as NK cells, T cells, macrophages and other related anti-cancer cells in the PCa microenvironment can further reveal the cause of immunotherapy failure and provide guidelines for PCa immunotherapy.

In our study, we identified and quantified 22 immune cells. It should be noted that there might be individual differences in the infiltration of immune cells, suggesting that when setting the medical reference interval of the infiltration proportion of immune cells, we need to include enough samples to reduce random errors. In general, there were 10 immune cells with significant infiltrating differences between groups; seven were significantly up-regulated and three were significantly down-regulated in PCa tissues. Three of the seven up-regulated immune cells were cells in the quiescent stage, suggesting that the initiation of these immune cells in the PCa microenvironment is inhibited. And immune cells that promote PCa progression, such as M2 macrophages, activated mast cells, neutrophil are also up-regulated. Under the dual effect, we hypothesized this may be one of the reasons for the failure of PCa immunotherapy (47).

In the *Results* section, we divided 22 immune cells into two types: adaptive and innate immune cells. Among the adaptive immune cells, the highest infiltration in normal prostate tissues was CD8+ T cells, while plasma cells had the most elevated infiltration in PCa tissues. The immune cell with the most significant differential infiltration between groups was naive B cells. For innate immune cells, the highest infiltrating cell in normal tissues was resting mast cells, and in PCa tissue was activated NK cells. Resting mast cells were the immune cell with the most significant infiltrating difference among innate immune cells. Subsequently, we combined the immune cells and found that the infiltrating percentage of total T cells was the highest in both normal and cancer tissues. In the six total cell types (total B cells, total T cells, total NK cells, total macrophages, total dendritic cells, total mast cells), the infiltrating percentage of four cell types showed significant differences between groups including total B cells, total T cells, total NK cells, and total mast cells. The infiltration trend of total T cells and total mast cells was consistent, which were significantly reduced in PCa tissues. The infiltration of total B cells and total NK cells were both significantly increased. Interestingly, the elevated infiltration of B cells and NK cells was caused by a significant up-regulation of inactivated cell subtypes, suggesting that there might be an inhibiting factor in the PCa microenvironment that inhibits the activation and maturation of immune cells. Next, we separately analyzed the expression correlation of 22 immune cells in normal tissues and PCa tissues. The results showed that there was a correlation between most immune cells in normal tissues or cancer tissues. The immune cells in the PCa microenvironment may not be infiltrated individually but cooperate with other immune cells. The TMB analysis and gene mutation analysis support this hypothesis; five subtypes of T cells together varied with the TMB significantly. The TMB test is the most important guideline for the implementation of immunotherapy, which also mainly explains that low TMB is one of the most important reasons for immunotherapy failure (43). Our results reveal the synergistic infiltrating effects of five T cells’ subtypes in PCa tissues with different TMB scores. In consequence, the infiltration of these five immune cells can be reversely used for preliminary evaluation of the TMB, and TMB may also be one of the mechanisms related to the infiltrating correlation of other immune cells. Most PCa patients have low TMB, and the insufficient T cell infiltration caused by low TMB might be a reason for ineffective immunotherapy for PCa. Consistent with TMB analysis results, the single gene mutation analysis support that genome changes are an important mechanism of immune cell infiltration abnormalities. Also, the mutations of the same immune gene will simultaneously change the infiltration of multiple immune cells, and it might be another reason why different immune cells had infiltrating correlation.

Gleason grade score is used as an indicator for PCa progression, and higher grade score signifies higher malignancy. The results revealed that the infiltration of 12 kinds of immune cells significantly changed together with the Gleason grades, suggesting that there is a different infiltration of immune cells in different Gleason grades of PCa. However, not all infiltration trends are linear, and each immune cell needs to be analyzed separately. Therefore, when we analyzed the reference value of immune cells’ proportion, we need to combine the information of different cancer grades and ages to give an accurate reference range. As mentioned previously, each immune cell works not alone but in concert, and the pathogenesis of prostate cancer is complicated, so we believe that the study of a single type of immune cell cannot primely reveal the role of the immune system in the progression and development of prostate cancer. However, the diagnostic effect and survival prediction ability of multiple immune cells on PCa are not known.

Therefore, we integrated the immune cells with significant differences between groups and used a nomogram to evaluate the prediction efficiency of these 10 immune cells. The results showed that all 10 immune cells and age have an impact on the five- and 10-year survival predictions. In addition, the prediction model showed a favorable efficacy. These results suggested that we should not use an index of an immune cell alone in clinical testing, but should comprehensively analyze multiple immune cells index of PCa patients in various aspects.

Of note, we also found that high infiltration of M1 macrophages and neutrophils were associated with poor prognosis. Researchers have found that high expression of neutrophils in peripheral blood is associated with poor prognosis in PCa (49, 50). Our study reveals that neutrophils infiltrating into PCa tissues have the same prognostic effect, which is also a potential therapeutic target. In previous studies, M1 macrophages were often known to inhibit cancer progression. Zhang et al. reported that low infiltration of M1 macrophages was associated with poor progress of the PCa patients in TCGA cohort (51). Of note, neither the source data for deconvolution nor the data processing and normalization method between Zhang et al. and the present study was different. These different data processing procedures may cause different samples to be included, which had a certain impact on the results. In our study, we combined the CIBERSORT results from TCGA cohort and GSE16560 cohort, and the survival analysis revealed that high infiltration of M1 macrophages in PCa tissues was found to reduce patient survival time. There are two possible reasons for this opposite conclusion: one is that our model does not include enough samples and leads to result shifts, and another one is that M1 macrophages infiltrating into the PCa microenvironment are polarized into M2 macrophages (50). However, the mechanism needs to be further studied by experiments.

To sum up, our study used extensive samples’ data to perform immunocytotyping and quantification of normal prostate and PCa tissues, which helps us to further understand the differences between PCa immune microenvironment and other cancers and also offer clues for exploring the validation of PCa immunotherapy. The potential therapeutic targets implicated in the present study may provide new insightful for clinical treatment.

## Data Availability Statement

Publicly available datasets were analyzed in this study. This data can be found here: GSE41969,GSE44353,GSE6606,GSE6287,GSE64333,GSE7055,GSE68555,GSE68882, GSE70768,GSE71783,GSE1431,GSE16120, GSE15484,GSE16560,GSE2443,GSE26242, GSE26910,GSE32448,GSE32571,GSE35988, GSE5132, GSE71016, GSE38241, GSE29079, GSE7220, GSE77959, TCGA-PRAD(https://portal.gdc.cancer.gov/).

## Ethics Statement

Ethical review and approval was not required for the study on human participants in accordance with the local legislation and institutional requirements. Written informed consent for participation was not required for this study in accordance with the national legislation and the institutional requirements.

## Author Contributions

ZW designed and conducted the experiments, performed data analysis, and wrote the manuscript. HC, WL, HZ, and GL performed experiments and data analysis. FZ and FD designed the study, interpreted the data, wrote the manuscript, and approved the final version of the manuscript for publication. All authors contributed to the article and approved the submitted version.

## Funding

This work was supported financially by the National Natural Science Foundation of China (Grant No. 81772761, 81672540, 81472407); Science and Technology Foundation of Guangzhou in China (Grant No. 201607010351, 201707010303), President Foundation of The Fifth Affiliated Hospital, Southern Medical University (YZ2017ZD002). Funding bodies did not have any influence on the design of the study and data collection, analysis, and interpretation of data or in writing the manuscript.

## Conflict of Interest

The authors declare that the research was conducted in the absence of any commercial or financial relationships that could be construed as a potential conflict of interest.

## Abbreviation

PCa, prostate cancer; AUC, The Area Under Curve; TCGA, The Cancer Genome Atlas; GEO, Gene Expression Omnibus; ADT, Androgen Deprivation Therapy; TIICs, Tumor-infiltrating immune cells; KM, Kaplan-Meier; TILs, tumor-infiltrating lymphocytes; OS, overall survival; ICB, immune checkpoint; TMB, Tumor Mutational Burden; SNV, Single nucleotide variants; iNKT, Invariant Natural Killer T cells; Tregs, regulatory T cells; NK cells, Natural Killer cells.
