# Supplementary material for: The Landscape of Immune Cells Infiltrating in Prostate Cancer
Source: Front Oncol. 2020 Oct 29;10:517637. doi: 10.3389/fonc.2020.517637 (PMC7658630; doi:10.3389/fonc.2020.517637)
Supplement: Supplementary file 1 [file DataSheet_1.pdf]

## Supplementary Figure 1

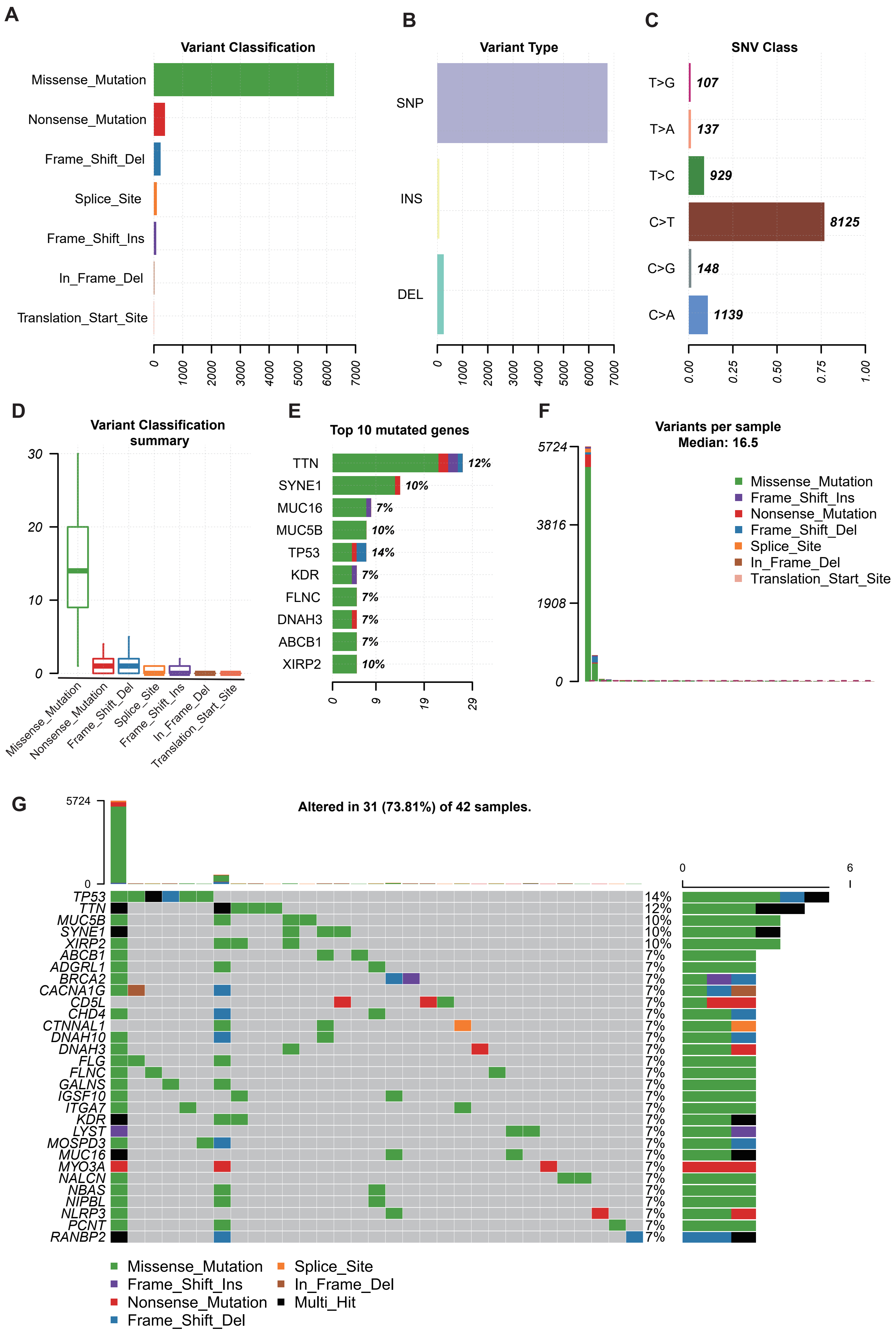

**Supplementary Figure 1.** Single Nucleotide Variants type ,classification and mutant genes in PCa patients(n=42). (A)Total number of seven variants classification. (B)Total number of three variants types. (C)Frequency of base mutation. Sum of 6 SNV class frequency equal to 100%. Number beside column was total mutation events. (D-F)Variants classification(D) and frequency(E) per samples. Top 10 mutated genes(F). D, E and F share the same legend. (G)Mutation frequency matrix of top 30 mutant genes.(Note: Variant annotated as Multi_Hit are those genes which are mutated more than once in the same sample; No Translation_Start_Site variant occurred in top 30 mutant gene)
