# Supplementary material for: The Landscape of Immune Cells Infiltrating in Prostate Cancer
Source: Front Oncol. 2020 Oct 29;10:517637. doi: 10.3389/fonc.2020.517637 (PMC7658630; doi:10.3389/fonc.2020.517637)
Supplement: Supplementary file 2 [file DataSheet_2.pdf]

Supplementary Figure 2

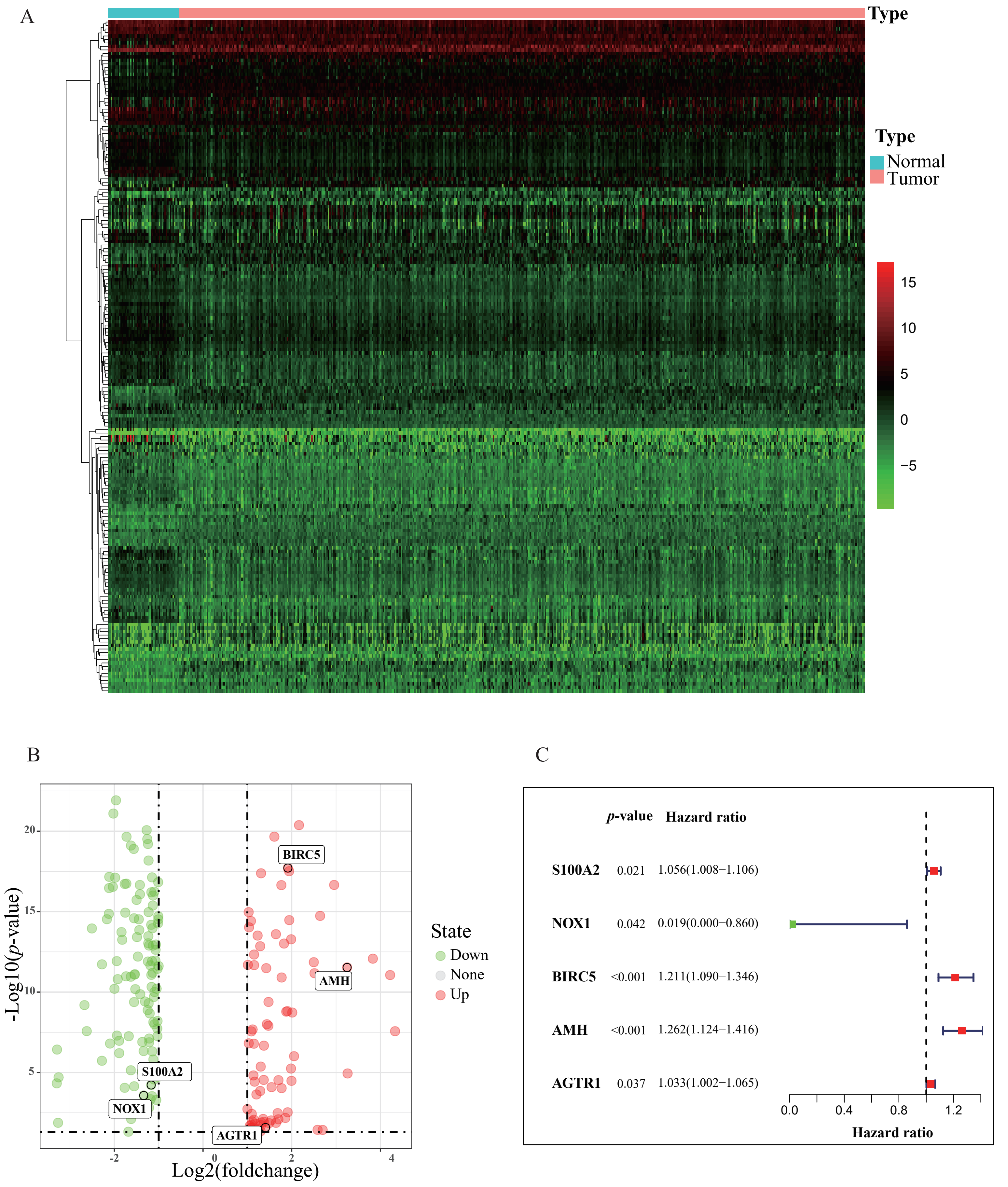

Supplementary Figure 2: The identification of five key immune genes. (A)Heatmap of 193 differential immune gene
(B)Volcano plot of differential immune genes. (C)Univariate Cox regression of immune genes.
