# Supplementary material for: The Landscape of Immune Cells Infiltrating in Prostate Cancer
Source: Front Oncol. 2020 Oct 29;10:517637. doi: 10.3389/fonc.2020.517637 (PMC7658630; doi:10.3389/fonc.2020.517637)
Supplement: Supplementary file 3 [file Table_1.docx]

**Supplementary Table 1. All samples ID and samples applied for each analysis**

1. **727 samples used for infiltrating differential and correlation analysis(Fig1, Fig2, Fig3, Fig4)**

| **Group** | **Dataset** | **Subset** | **Note** |
| --- | --- | --- | --- |
| **Normal** | TCGA | TCGA-EJ-7315-11A-01R-2118-07, TCGA-HC-7752-11A-01R-2118-07, TCGA-EJ-7782-11A-01R-2118-07, TCGA-EJ-7783-11A-01R-2118-07 |  |
|  | GSE26910 | GSM662756, GSM662758, GSM662760, GSM662762, GSM662764, GSM662766 |  |
|  | GSE32448 | GSM802826, GSM802828, GSM802838, GSM802874, GSM802876 |  |
|  | GSE2443 | GSM45730, GSM45847, GSM45848, GSM45849, GSM45850, GSM45851, GSM45852, GSM45853, GSM45854 |  |
|  | GSE5132 | GSM115775, GSM115781, GSM115782, GSM115784 |  |
|  | GSE71016 | GSM1825572, GSM1825582, GSM1825598, GSM1825601 |  |
|  | GSE15484 | GSM388476, GSM388489, GSM388490, GSM388492 |  |
|  | GSE32571 | GSM807247, GSM807248, GSM807294, GSM807295, GSM807321 |  |
|  | GSE38241 | GSM937289, GSM937293, GSM937294, GSM937295, GSM937297, GSM937298, GSM937300, GSM937302, GSM937307 |  |
|  | GSE41969 | GSM1028927, GSM1029006, GSM1029014, GSM1029031, GSM1029072 |  |
|  | GSE62872 | GSM1535057, GSM1535058, GSM1535064, GSM1535076, GSM1535077, GSM1535078, GSM1535079, GSM1535081, GSM1535082, GSM1535085, GSM1535086, GSM1535087, GSM1535095, GSM1535096, GSM1535098, GSM1535100, GSM1535101, GSM1535104, GSM1535105, GSM1535107, GSM1535108, GSM1535111, GSM1535114, GSM1535120, GSM1535121, GSM1535124, GSM1535129, GSM1535130, GSM1535131, GSM1535133, GSM1535135, GSM1535138, GSM1535140, GSM1535141, GSM1535142, GSM1535146, GSM1535147, GSM1535149, GSM1535151, GSM1535153, GSM1535156, GSM1535157, GSM1535159, GSM1535160, GSM1535161, GSM1535162, GSM1535164, GSM1535165, GSM1535168, GSM1535169, GSM1535170, GSM1535173, GSM1535175, GSM1535176, GSM1535177, GSM1535178, GSM1535184, GSM1535185, GSM1535187, GSM1535190, GSM1535191, GSM1535193, GSM1535194, GSM1535195, GSM1535196, GSM1535197, GSM1535199, GSM1535201, GSM1535203, GSM1535204, GSM1535205, GSM1535208, GSM1535212, GSM1535214, GSM1535216 |  |
|  | GSE70768 | GSM1817834, GSM1817835, GSM1817836, GSM1817837, GSM1817839, GSM1817842, GSM1817843, GSM1817844, GSM1817846, GSM1817849, GSM1817850, GSM1817853, GSM1817854, GSM1817855, GSM1817856, GSM1817857, GSM1817860, GSM1817862, GSM1817863, GSM1817864, GSM1817865, GSM1817867, GSM1817869, GSM1817870, GSM1817871, GSM1817872, GSM1817874, GSM1817875, GSM1817876, GSM1817877, GSM1817878, GSM1817879, GSM1817883, GSM1817885, GSM1817888, GSM1817890, GSM1817893, GSM1817896, GSM1817897, GSM1817898, GSM1817899, GSM1817900, GSM1817901, GSM1817902, GSM1817904 |  |
|  | GSE29079 | GSM720516, GSM720529, GSM720546 |  |
|  | GSE72220 | GSM1857897, GSM1857901, GSM1857942, GSM1857948, GSM1857955 |  |
|  | GSE77959 | GSM2062420, GSM2062422, GSM2062424, GSM2062426, GSM2062430, GSM2062438, GSM2062442 |  |
| **Tumor** | TCGA | TCGA-J4-A67N-01A-11R-A30B-07, TCGA-ZG-A9N3-01A-11R-A41O-07, TCGA-ZG-A9MC-01A-31R-A41O-07, TCGA-XK-AAIV-01A-11R-A41O-07, TCGA-ZG-A9KY-01A-11R-A41O-07, TCGA-G9-6498-01A-12R-A311-07, TCGA-HC-7079-01A-11R-1965-07, TCGA-ZG-A9LN-01A-11R-A41O-07, TCGA-G9-7523-01A-11R-2263-07, TCGA-H9-A6BX-01A-31R-A311-07, TCGA-EJ-A65B-01A-12R-A30B-07, TCGA-HC-A9TH-01A-11R-A41O-07, TCGA-XK-AAIW-01A-11R-A41O-07, TCGA-J9-A8CP-01A-11R-A352-07, TCGA-ZG-A9M4-01A-11R-A41O-07, TCGA-KK-A7B0-01A-11R-A32O-07, TCGA-HC-A6AS-01A-11R-A30B-07, TCGA-CH-5751-01A-11R-1580-07, TCGA-HC-A9TE-01A-11R-A41O-07, TCGA-G9-7521-01A-11R-2263-07, TCGA-EJ-5531-01A-01R-1580-07, TCGA-ZG-A9LS-01A-12R-A41O-07, TCGA-CH-5754-01A-11R-1580-07, TCGA-V1-A8MF-01A-11R-A36G-07, TCGA-M7-A723-01A-12R-A32O-07, TCGA-KK-A8I4-01A-11R-A36G-07, TCGA-V1-A9O9-01A-11R-A41O-07, TCGA-YL-A9WY-01A-11R-A41O-07, TCGA-KK-A59V-01A-11R-A29R-07, TCGA-VP-A87C-01A-11R-A352-07, TCGA-KK-A7B2-01A-12R-A32O-07, TCGA-VN-A88I-01A-11R-A352-07, TCGA-G9-6379-01A-11R-A31N-07, TCGA-G9-6347-01A-11R-A31N-07, TCGA-EJ-7328-01A-31R-2118-07, TCGA-J4-A6G1-01A-11R-A311-07, TCGA-KK-A7AV-01A-11R-A32O-07, TCGA-HC-8266-01A-11R-2263-07, TCGA-EJ-8470-01A-11R-2403-07, TCGA-V1-A8MU-01A-11R-A37L-07, TCGA-4L-AA1F-01A-11R-A41O-07, TCGA-V1-A9O5-01A-11R-A41O-07, TCGA-J4-A67M-01A-11R-A30B-07, TCGA-XJ-A9DI-01A-11R-A37L-07 |  |
|  | GSE26910 | GSM662757, GSM662759, GSM662763, GSM662765, GSM662767 |  |
|  | GSE32448 | GSM802845, GSM802847, GSM802857, GSM802859, GSM802861, GSM802869, GSM802873 |  |
|  | GSE2443 | GSM45857, GSM45858, GSM45859, GSM45860, GSM45861, GSM45863, GSM45864, GSM45865 |  |
|  | GSE1431 | GSM23762, GSM23763, GSM23770, GSM23771, GSM23773, GSM23778, GSM23804, GSM23808, GSM23809, GSM23813, GSM23825, GSM23829, GSM23830, GSM23832, GSM23834, GSM23835, GSM23839, GSM23840, GSM23848 |  |
|  | GSE6606 | GSM153036, GSM153054, GSM153060, GSM153062, GSM153064, GSM153067, GSM153068, GSM153071, GSM153072, GSM153073, GSM153074, GSM153080, GSM153082, GSM153084, GSM153087, GSM153095, GSM153097, GSM153098, GSM153099, GSM153104, GSM153110, GSM153112, GSM153113, GSM153114, GSM187535, GSM152939, GSM152942, GSM152959, GSM152973, GSM152988, GSM152990, GSM187527 |  |
|  | GSE15484 | GSM388474, GSM388475, GSM388479, GSM388480, GSM388487, GSM388496, GSM388512, GSM388513, GSM388518, GSM388528 |  |
|  | GSE16120 | GSM355113, GSM355116, GSM355117, GSM355129, GSM355146, GSM355149, GSM355153, GSM355154, GSM355155, GSM355156, GSM355158 |  |
|  | GSE16560 | GSM416246, GSM416248, GSM416253, GSM416255, GSM416266, GSM416269, GSM416294, GSM416302, GSM416310, GSM416317, GSM416364, GSM416371, GSM416380, GSM416382, GSM416393, GSM416395, GSM416398, GSM416405, GSM416406, GSM416420, GSM416427, GSM416428, GSM416439, GSM416458, GSM416471, GSM416472, GSM416480, GSM416483, GSM416488, GSM416493, GSM416504, GSM416511, GSM416512, GSM416520 |  |
|  | GSE26242 | GSM644090, GSM644097, GSM644110, GSM644146, GSM644159, GSM644175, GSM644176 |  |
|  | GSE32571 | GSM807271, GSM807276, GSM807303, GSM807305, GSM807315 |  |
|  | GSE35988 | GSM878788, GSM878793, GSM878795, GSM878797, GSM878799, GSM878800, GSM878801, GSM878811, GSM878825, GSM878832, GSM878913, GSM878951, GSM878953, GSM878984, GSM878988 |  |
|  | GSE41969 | GSM1028293, GSM1028404, GSM1028516, GSM1028686, GSM1028775, GSM1028844 |  |
|  | GSE44353 | GSM1083657, GSM1083658, GSM1083659, GSM1083660, GSM1083661, GSM1083662, GSM1083663, GSM1083664, GSM1083666, GSM1083668, GSM1083669, GSM1083670, GSM1083671, GSM1083672, GSM1083676, GSM1083678, GSM1083680, GSM1083681, GSM1083682, GSM1083683, GSM1083684, GSM1083686, GSM1083693, GSM1083694, GSM1083697, GSM1083699, GSM1083701, GSM1083709, GSM1083710, GSM1083712, GSM1083713, GSM1083715, GSM1083721, GSM1083724, GSM1083725, GSM1083726, GSM1083727, GSM1083728, GSM1083731, GSM1083732, GSM1083733, GSM1083736, GSM1083740, GSM1083741, GSM1083748, GSM1083752, GSM1083753, GSM1083758, GSM1083761, GSM1083762, GSM1083763, GSM1083764, GSM1083769, GSM1083771, GSM1083772, GSM1083774, GSM1083776, GSM1083780, GSM1083781, GSM1083783, GSM1083784, GSM1083785, GSM1083787, GSM1083789, GSM1083790, GSM1083791, GSM1083792, GSM1083793, GSM1083794, GSM1083795, GSM1083798, GSM1083802, GSM1083803, GSM1083808, GSM1083813, GSM1083814, GSM1083815, GSM1083816, GSM1083817, GSM1083821, GSM1083823, GSM1083828, GSM1083833, GSM1083836, GSM1083837, GSM1083838, GSM1083840, GSM1083843, GSM1083845, GSM1083851, GSM1083855, GSM1083856 |  |
|  | GSE62872 | GSM1534794, GSM1534799, GSM1534800, GSM1534806, GSM1534809, GSM1534810, GSM1534816, GSM1534823, GSM1534832, GSM1534837, GSM1534838, GSM1534840, GSM1534841, GSM1534843, GSM1534848, GSM1534850, GSM1534851, GSM1534863, GSM1534868, GSM1534870, GSM1534871, GSM1534873, GSM1534875, GSM1534876, GSM1534882, GSM1534884, GSM1534886, GSM1534887, GSM1534888, GSM1534889, GSM1534892, GSM1534893, GSM1534898, GSM1534901, GSM1534902, GSM1534903, GSM1534907, GSM1534908, GSM1534912, GSM1534914, GSM1534926, GSM1534930, GSM1534932, GSM1534933, GSM1534934, GSM1534941, GSM1534945, GSM1534946, GSM1534957, GSM1534958, GSM1534959, GSM1534961, GSM1534971, GSM1534972, GSM1534973, GSM1534974, GSM1534978, GSM1534981, GSM1534984, GSM1534985, GSM1534989, GSM1534992, GSM1534994, GSM1534999, GSM1535001, GSM1535005, GSM1535007, GSM1535014, GSM1535016, GSM1535021, GSM1535023, GSM1535025, GSM1535027, GSM1535028, GSM1535033, GSM1535034, GSM1535041, GSM1535046, GSM1535048, GSM1535049, GSM1535051, GSM1535056 |  |
|  | GSE68555 | GSM1675050, GSM1675053, GSM1675079, GSM1675082, GSM1675088, GSM1675094, GSM1675103, GSM1675106, GSM1675124, GSM1675158, GSM1675161, GSM1675167, GSM1675170, GSM1675173, GSM1675185, GSM1675194, GSM1675202, GSM1675211, GSM1675229, GSM1675235, GSM1675238, GSM1675243, GSM1675252, GSM1675255, GSM1675273, GSM1675279, GSM1675285, GSM1675294, GSM1675297, GSM1675307, GSM1675310, GSM1675313, GSM1675316, GSM1675334, GSM1675340, GSM1675346, GSM1675378, GSM1675384, GSM1675387, GSM1675390, GSM1675405, GSM1675423 |  |
|  | GSE68882 | GSM1685006, GSM1685009, GSM1685011, GSM1685012, GSM1685016, GSM1685022, GSM1685024, GSM1685034 |  |
|  | GSE70768 | GSM1817707, GSM1817708, GSM1817710, GSM1817711, GSM1817712, GSM1817713, GSM1817716, GSM1817718, GSM1817719, GSM1817722, GSM1817729, GSM1817735, GSM1817737, GSM1817746, GSM1817750, GSM1817763, GSM1817770, GSM1817776, GSM1817777, GSM1817778, GSM1817786, GSM1817790, GSM1817791, GSM1817803, GSM1817807, GSM1817813, GSM1817814, GSM1817815, GSM1817816, GSM1817821, GSM1817824, GSM1817825, GSM1817826, GSM1817827, GSM1817831 |  |
|  | GSE7055 | GSM160345, GSM160346, GSM160347, GSM160348, GSM160351, GSM160353, GSM160354, GSM160355, GSM160357, GSM160360, GSM160361, GSM160362, GSM160364, GSM160365, GSM160366, GSM160368, GSM160369, GSM160370, GSM160373, GSM160375, GSM160376, GSM160377, GSM160378, GSM160379, GSM160382, GSM160383, GSM160384, GSM160385, GSM160386, GSM160387, GSM160388, GSM160389, GSM160390, GSM160391, GSM160396, GSM160397, GSM160398, GSM160399, GSM160400 |  |
|  | GSE64333 | GSM1568944, GSM1568945, GSM1568950, GSM1568959, GSM1568968, GSM1568970, GSM1568978, GSM1568985, GSM1568987, GSM1568991, GSM1568993, GSM1568997, GSM1568999, GSM1569001, GSM1569015 |  |
|  | GSE71783 | GSM1845172, GSM1845173, GSM1845175, GSM1845178, GSM1845179, GSM1845190, GSM1845191, GSM1845196, GSM1845197, GSM1845237, GSM1845239, GSM1845241, GSM1845243, GSM1845244, GSM1845245, GSM1845246, GSM1845247, GSM1845248, GSM1845250, GSM1845251, GSM1845253 |  |

1. **203 samples used for Gleason grade and immune cells correlation analysis(Fig.5)**

| **Group** | **Dataset** | **Subset** | **Note** |
| --- | --- | --- | --- |
| **Tumor** | GSE44353 | GSM1083657, GSM1083658, GSM1083659, GSM1083660, GSM1083661, GSM1083662, GSM1083663, GSM1083664, GSM1083666, GSM1083668, GSM1083669, GSM1083670, GSM1083671, GSM1083672, GSM1083676, GSM1083678, GSM1083680, GSM1083681, GSM1083682, GSM1083683, GSM1083684, GSM1083686, GSM1083693, GSM1083694, GSM1083697, GSM1083699, GSM1083701, GSM1083709, GSM1083710, GSM1083712, GSM1083713, GSM1083715, GSM1083721, GSM1083724, GSM1083725, GSM1083726, GSM1083727, GSM1083728, GSM1083731, GSM1083732, GSM1083733, GSM1083736, GSM1083740, GSM1083741, GSM1083748, GSM1083752, GSM1083753, GSM1083758, GSM1083761, GSM1083762, GSM1083763, GSM1083764, GSM1083769, GSM1083772, GSM1083774, GSM1083776, GSM1083780, GSM1083781, GSM1083783, GSM1083784, GSM1083785, GSM1083787, GSM1083789, GSM1083790, GSM1083791, GSM1083792, GSM1083793, GSM1083794, GSM1083795, GSM1083798, GSM1083802, GSM1083803, GSM1083808, GSM1083813, GSM1083814, GSM1083815, GSM1083816, GSM1083817, GSM1083821, GSM1083823, GSM1083828, GSM1083833, GSM1083836, GSM1083837, GSM1083838, GSM1083840, GSM1083843, GSM1083845, GSM1083851, GSM1083855, GSM1083856 |  |
|  | GSE7055 | GSM160345, GSM160346, GSM160347, GSM160348, GSM160351, GSM160353, GSM160354, GSM160355, GSM160357, GSM160360, GSM160361, GSM160362, GSM160364, GSM160365, GSM160366, GSM160368, GSM160369, GSM160370, GSM160373, GSM160375, GSM160376, GSM160377, GSM160378, GSM160379, GSM160382, GSM160383, GSM160384, GSM160385, GSM160386, GSM160387, GSM160388, GSM160389, GSM160390, GSM160391, GSM160396, GSM160397, GSM160398, GSM160399, GSM160400 |  |
|  | GSE68555 | GSM1675050, GSM1675053, GSM1675079, GSM1675082, GSM1675088, GSM1675094, GSM1675103, GSM1675106, GSM1675124, GSM1675158, GSM1675161, GSM1675167, GSM1675170, GSM1675173, GSM1675185, GSM1675194, GSM1675202, GSM1675211, GSM1675229, GSM1675235, GSM1675238, GSM1675243, GSM1675252, GSM1675255, GSM1675273, GSM1675279, GSM1675285, GSM1675294, GSM1675297, GSM1675307, GSM1675310, GSM1675313, GSM1675316, GSM1675334, GSM1675340, GSM1675346, GSM1675378, GSM1675384, GSM1675387, GSM1675390, GSM1675405, GSM1675423 |  |
|  | GSE70768 | GSM1817707, GSM1817711, GSM1817712, GSM1817713, GSM1817719, GSM1817722, GSM1817729, GSM1817735, GSM1817737, GSM1817746, GSM1817750, GSM1817763, GSM1817770, GSM1817776, GSM1817777, GSM1817778, GSM1817786, GSM1817790, GSM1817791, GSM1817803, GSM1817807, GSM1817813, GSM1817814, GSM1817815, GSM1817816, GSM1817821, GSM1817824, GSM1817825, GSM1817826, GSM1817827, GSM1817831 |  |

**(3) 486 samples used for TMB related functional enrichment analysis (Fig.6A, B, C)**

| **Group** | **Dataset** | **Subset** | **Note** |
| --- | --- | --- | --- |
| **Tumor** | TCGA | TCGA-EJ-A65G-01A-21R-A29R-07, TCGA-YL-A8SR-01B-11R-A37L-07, TCGA-QU-A6IL-01A-11R-A31N-07, TCGA-G9-6329-01A-13R-1965-07, TCGA-KK-A7AU-01A-11R-A32O-07, TCGA-XK-AAJT-01A-11R-A41O-07, TCGA-EJ-A6RC-01A-11R-A32O-07, TCGA-EJ-7784-01A-11R-2118-07, TCGA-EJ-5506-01A-01R-1580-07, TCGA-EJ-7218-01B-11R-A32O-07, TCGA-Y6-A8TL-01A-21R-A37L-07, TCGA-HC-7748-01A-11R-2118-07, TCGA-2A-A8W1-01A-11R-A37L-07, TCGA-VN-A88O-01A-11R-A352-07, TCGA-HC-A6HY-01A-11R-A31N-07, TCGA-KC-A4BR-01A-32R-A32Y-07, TCGA-YL-A9WJ-01A-11R-A37L-07, TCGA-VN-A88N-01A-11R-A36G-07, TCGA-HC-8261-01A-11R-2263-07, TCGA-G9-6379-01A-11R-A31N-07, TCGA-G9-6343-01A-21R-1965-07, TCGA-YL-A8SB-01A-31R-A37L-07, TCGA-J4-AAU2-01A-11R-A41O-07, TCGA-KK-A8I4-01A-11R-A36G-07, TCGA-V1-A9OA-01A-11R-A41O-07, TCGA-KK-A8IF-01A-11R-A36G-07, TCGA-KK-A7B1-01A-11R-A32O-07, TCGA-G9-7522-01A-11R-2263-07, TCGA-J4-A67N-01A-11R-A30B-07, TCGA-EJ-8469-01A-11R-2403-07, TCGA-EJ-A46D-01A-21R-A32Y-07, TCGA-VP-A87E-01A-31R-A352-07, TCGA-HC-8258-01B-05R-2302-07, TCGA-G9-6370-01A-11R-1789-07, TCGA-CH-5763-01A-11R-1580-07, TCGA-EJ-A7NF-01A-11R-A33R-07, TCGA-KC-A4BN-01A-61R-A250-07, TCGA-J4-A83M-01A-11R-A352-07, TCGA-EJ-5517-01A-01R-1580-07, TCGA-2A-AAYF-01A-11R-A41O-07, TCGA-YL-A8HK-01A-11R-A36G-07, TCGA-HC-7736-01A-11R-2118-07, TCGA-V1-A9O9-01A-11R-A41O-07, TCGA-CH-5789-01A-11R-1580-07, TCGA-HC-A8D1-01A-11R-A36G-07, TCGA-EJ-8468-01A-21R-2403-07, TCGA-V1-A9ZK-01A-11R-A41O-07, TCGA-KC-A7FE-01A-12R-A33R-07, TCGA-G9-6336-01A-11R-1789-07, TCGA-EJ-A7NK-01A-12R-A352-07, TCGA-2A-AAYO-01A-11R-A41O-07, TCGA-VP-A872-01A-11R-A352-07, TCGA-HC-7819-01A-11R-2118-07, TCGA-HC-8259-01A-11R-2263-07, TCGA-ZG-A9L2-01A-31R-A41O-07, TCGA-G9-6353-01A-11R-1965-07, TCGA-HC-7821-01A-12R-2118-07, TCGA-V1-A9OX-01A-11R-A41O-07, TCGA-V1-A8MG-01A-11R-A36G-07, TCGA-EJ-A46I-01A-12R-A26U-07, TCGA-G9-A9S7-01A-11R-A41O-07, TCGA-EJ-AB20-01A-12R-A41O-07, TCGA-V1-A8X3-01A-11R-A37L-07, TCGA-VN-A88I-01A-11R-A352-07, TCGA-HC-7740-01A-11R-2118-07, TCGA-YL-A9WX-01A-21R-A41O-07, TCGA-HC-8266-01A-11R-2263-07, TCGA-EJ-A46B-01A-31R-A250-07, TCGA-KK-A59Y-01A-11R-A26U-07, TCGA-EJ-8470-01A-11R-2403-07, TCGA-XK-AAIR-01A-11R-A41O-07, TCGA-HC-7740-01B-04R-2302-07, TCGA-G9-7509-01A-11R-A41O-07, TCGA-HC-7817-01B-11R-A29R-07, TCGA-ZG-A8QZ-01A-11R-A37L-07, TCGA-G9-6356-01A-11R-1789-07, TCGA-G9-6333-01A-12R-1965-07, TCGA-EJ-7794-01A-11R-2118-07, TCGA-J4-8200-01A-11R-A29R-07, TCGA-ZG-A9L4-01A-11R-A41O-07, TCGA-KK-A6E1-01A-11R-A311-07, TCGA-EJ-5522-01A-01R-1580-07, TCGA-VP-A87C-01A-11R-A352-07, TCGA-EJ-7786-01A-11R-2118-07, TCGA-CH-5766-01A-11R-1580-07, TCGA-J9-A8CN-01A-11R-A352-07, TCGA-YL-A8SJ-01B-11R-A37L-07, TCGA-HC-A8D0-01A-11R-A36G-07, TCGA-EJ-A7NG-01A-31R-A33R-07, TCGA-HC-8260-01A-11R-2263-07, TCGA-KK-A8I7-01A-21R-A36G-07, TCGA-EJ-5510-01A-01R-1580-07, TCGA-KK-A7B2-01A-12R-A32O-07, TCGA-G9-6364-01A-21R-1789-07, TCGA-G9-6366-01A-11R-2118-07, TCGA-KK-A8IG-01A-11R-A36G-07, TCGA-V1-A8ML-01A-11R-A37L-07, TCGA-G9-6385-01A-11R-1789-07, TCGA-G9-6347-01A-11R-A31N-07, TCGA-J4-A67T-01A-11R-A311-07, TCGA-M7-A721-01A-12R-A32O-07, TCGA-M7-A723-01A-12R-A32O-07, TCGA-HC-7745-01A-11R-2118-07, TCGA-V1-A8MM-01A-11R-A37L-07, TCGA-HC-7081-01A-11R-1965-07, TCGA-CH-5745-01A-11R-1580-07, TCGA-YL-A9WL-01A-11R-A41O-07, TCGA-EJ-5516-01A-01R-1580-07, TCGA-CH-5738-01A-11R-1580-07, TCGA-G9-7519-01A-11R-2263-07, TCGA-G9-6384-01A-11R-1789-07, TCGA-HC-A6AQ-01A-11R-A30B-07, TCGA-EJ-7793-01A-31R-2263-07, TCGA-HC-7752-01A-11R-2118-07, TCGA-EJ-5508-01A-02R-1580-07, TCGA-KK-A7AQ-01A-11R-A33R-07, TCGA-EJ-7315-01A-31R-2118-07, TCGA-EJ-7312-01B-21R-A32O-07, TCGA-EJ-A7NH-01A-12R-A33R-07, TCGA-EJ-A65B-01A-12R-A30B-07, TCGA-CH-5764-01A-21R-1580-07, TCGA-VN-A88M-01A-11R-A352-07, TCGA-J4-A67L-01A-11R-A30B-07, TCGA-EJ-5530-01A-01R-1580-07, TCGA-4L-AA1F-01A-11R-A41O-07, TCGA-KC-A7F6-01A-11R-A33R-07, TCGA-EJ-7792-01A-11R-2118-07, TCGA-KK-A8I8-01A-11R-A36G-07, TCGA-HC-A6AP-01A-11R-A30B-07, TCGA-YL-A8HL-01A-11R-A36G-07, TCGA-G9-6378-01A-11R-1789-07, TCGA-FC-7708-01A-11R-2118-07, TCGA-KK-A6E3-01A-21R-A30B-07, TCGA-G9-6342-01A-11R-1965-07, TCGA-HC-7230-01A-11R-2118-07, TCGA-J4-A67R-01A-21R-A30B-07, TCGA-EJ-5512-01A-01R-1580-07, TCGA-M7-A71Z-01A-12R-A32O-07, TCGA-G9-A9S0-01A-11R-A41O-07, TCGA-HC-8258-01A-11R-2263-07, TCGA-EJ-A8FO-01A-21R-A36G-07, TCGA-HC-7744-01A-11R-2118-07, TCGA-M7-A71Y-01A-22R-A32O-07, TCGA-EJ-A6RA-01A-11R-A33R-07, TCGA-CH-5741-01A-11R-1580-07, TCGA-YL-A9WK-01A-11R-A37L-07, TCGA-G9-6369-01A-21R-1965-07, TCGA-HC-7820-01A-11R-2118-07, TCGA-G9-6498-01A-12R-A311-07, TCGA-HC-7075-01A-11R-1965-07, TCGA-ZG-A9L6-01A-11R-A41O-07, TCGA-HC-8213-01A-11R-A29R-07, TCGA-CH-5740-01A-11R-1580-07, TCGA-G9-7523-01A-11R-2263-07, TCGA-CH-5771-01A-21R-1580-07, TCGA-HC-7818-01A-11R-2118-07, TCGA-YL-A8SI-01A-11R-A41O-07, TCGA-G9-6365-01A-11R-1789-07, TCGA-H9-A6BY-01A-11R-A30B-07, TCGA-J4-A67S-01A-11R-A30B-07, TCGA-G9-6332-01A-11R-1789-07, TCGA-EJ-5531-01A-01R-1580-07, TCGA-EJ-7785-01A-11R-2118-07, TCGA-H9-A6BX-01A-31R-A311-07, TCGA-V1-A8WN-01A-11R-A37L-07, TCGA-EJ-A8FP-01A-21R-A36G-07, TCGA-EJ-7331-01A-11R-2118-07, TCGA-G9-6377-01A-11R-1965-07, TCGA-X4-A8KS-01A-12R-A36G-07, TCGA-G9-7510-01A-11R-2263-07, TCGA-HC-8256-01A-11R-2263-07, TCGA-VP-A876-01A-11R-A352-07, TCGA-KK-A8I6-01A-11R-A36G-07, TCGA-2A-A8VV-01A-11R-A37L-07, TCGA-G9-6371-01A-11R-1789-07, TCGA-J4-A67K-01A-21R-A30B-07, TCGA-EJ-7125-01A-11R-1965-07, TCGA-V1-A9OY-01A-11R-A41O-07, TCGA-CH-5748-01A-11R-1580-07, TCGA-CH-5743-01A-21R-1580-07, TCGA-KK-A8IC-01A-11R-A36G-07, TCGA-XJ-A83H-01A-11R-A352-07, TCGA-J4-A83K-01A-11R-A352-07, TCGA-XJ-A9DI-01A-11R-A37L-07, TCGA-J4-A67O-01A-11R-A30B-07, TCGA-J4-A6M7-01A-11R-A31N-07, TCGA-XK-AAJU-01A-11R-A41O-07, TCGA-EJ-5515-01A-01R-1580-07, TCGA-J4-A6G3-01A-11R-A311-07, TCGA-SU-A7E7-01A-22R-A33R-07, TCGA-CH-5746-01A-11R-1580-07, TCGA-HC-7209-01A-11R-2118-07, TCGA-EJ-7797-01A-11R-2263-07, TCGA-KK-A6DY-01A-12R-A311-07, TCGA-EJ-7321-01A-31R-2263-07, TCGA-KK-A8IH-01A-11R-A36G-07, TCGA-HI-7169-01A-11R-2118-07, TCGA-EJ-7318-01B-11R-A32O-07, TCGA-MG-AAMC-01A-11R-A41O-07, TCGA-KK-A8IM-01A-11R-A36G-07, TCGA-YL-A8SP-01B-11R-A37L-07, TCGA-XJ-A9DQ-01A-11R-A37L-07, TCGA-EJ-7327-01A-11R-2118-07, TCGA-G9-6354-01A-11R-A311-07, TCGA-V1-A8WS-01A-11R-A37L-07, TCGA-J4-A67Q-01A-21R-A30B-07, TCGA-J4-A83L-01A-11R-A352-07, TCGA-G9-6351-01A-21R-1965-07, TCGA-J9-A8CK-01A-11R-A352-07, TCGA-EJ-7791-01A-11R-2118-07, TCGA-HC-A6HX-01A-11R-A31N-07, TCGA-KC-A4BL-01A-31R-A250-07, TCGA-EJ-5497-01A-02R-1580-07, TCGA-HC-A76X-01A-11R-A33R-07, TCGA-FC-A5OB-01A-11R-A29R-07, TCGA-QU-A6IP-01A-11R-A31N-07, TCGA-VN-A88R-01A-11R-A36G-07, TCGA-QU-A6IN-01A-11R-A31N-07, TCGA-VN-A88L-01A-11R-A352-07, TCGA-EJ-5501-01A-01R-1580-07, TCGA-EJ-5494-01A-01R-1580-07, TCGA-HC-7737-01A-11R-2118-07, TCGA-KK-A8IJ-01A-11R-A352-07, TCGA-KK-A6E5-01A-11R-A311-07, TCGA-EJ-5502-01A-01R-1580-07, TCGA-V1-A9Z8-01A-11R-A41O-07, TCGA-KK-A7AV-01A-11R-A32O-07, TCGA-FC-A8O0-01A-41R-A37L-07, TCGA-YL-A8HO-01A-11R-A36G-07, TCGA-ZG-A9MC-01A-31R-A41O-07, TCGA-HC-7750-01A-11R-2118-07, TCGA-HI-7170-01A-11R-2118-07, TCGA-HC-A6AS-01A-11R-A30B-07, TCGA-EJ-5542-01A-01R-1580-07, TCGA-HC-7212-01A-11R-2118-07, TCGA-J4-A83N-01A-11R-A352-07, TCGA-CH-5790-01A-11R-1580-07, TCGA-EJ-AB27-01A-11R-A41O-07, TCGA-XA-A8JR-01A-11R-A36G-07, TCGA-EJ-5509-01A-01R-1580-07, TCGA-VN-A943-01A-11R-A41O-07, TCGA-HC-7211-01A-11R-2118-07, TCGA-V1-A9O7-01A-21R-A41O-07, TCGA-V1-A9OF-01A-11R-A41O-07, TCGA-EJ-5527-01A-01R-1580-07, TCGA-HC-7747-01A-11R-2118-07, TCGA-ZG-A9LU-01A-11R-A41O-07, TCGA-2A-A8VO-01A-11R-A37L-07, TCGA-ZG-A9LS-01A-12R-A41O-07, TCGA-KK-A7AY-01A-11R-A33R-07, TCGA-CH-5762-01A-11R-1580-07, TCGA-YL-A9WI-01A-11R-A37L-07, TCGA-FC-7961-01A-11R-A29R-07, TCGA-EJ-A7NN-01A-11R-A33R-07, TCGA-VN-A88K-01A-11R-A352-07, TCGA-EJ-A65D-01A-11R-A30B-07, TCGA-2A-AAYU-01A-11R-A41O-07, TCGA-FC-A4JI-01A-11R-A250-07, TCGA-J4-A67M-01A-11R-A30B-07, TCGA-FC-A6HD-01A-11R-A31N-07, TCGA-HC-A6AO-01A-11R-A30B-07, TCGA-CH-5739-01A-11R-1580-07, TCGA-HC-8216-01A-11R-A29R-07, TCGA-J9-A8CL-01A-11R-A352-07, TCGA-VP-AA1N-01A-31R-A41O-07, TCGA-XK-AAIW-01A-11R-A41O-07, TCGA-ZG-A9L5-01A-12R-A41O-07, TCGA-HC-A6AL-01A-11R-A30B-07, TCGA-HC-A4ZV-01A-11R-A26U-07, TCGA-G9-6373-01A-11R-1789-07, TCGA-VP-A87J-01A-11R-A352-07, TCGA-ZG-A9NI-01A-11R-A41O-07, TCGA-KK-A6E4-01A-11R-A30B-07, TCGA-CH-5754-01A-11R-1580-07, TCGA-2A-A8VX-01A-11R-A37L-07, TCGA-HC-A48F-01A-11R-A250-07, TCGA-V1-A9O5-06A-11R-A41O-07, TCGA-Y6-A9XI-01A-11R-A41O-07, TCGA-HC-7210-01A-11R-2118-07, TCGA-KK-A6E2-01A-11R-A311-07, TCGA-KK-A5A1-01A-11R-A29R-07, TCGA-V1-A9ZI-01A-11R-A41O-07, TCGA-G9-A9S4-01A-11R-A41O-07, TCGA-KK-A7B0-01A-11R-A32O-07, TCGA-J4-8198-01A-11R-2263-07, TCGA-EJ-5511-01A-01R-1580-07, TCGA-EJ-5495-01A-01R-1580-07, TCGA-EJ-7782-01A-11R-2118-07, TCGA-XK-AAJP-01A-11R-A41O-07, TCGA-HC-A76W-01A-11R-A33R-07, TCGA-EJ-A7NM-01A-21R-A33R-07, TCGA-HC-A8CY-01A-11R-A36G-07, TCGA-KK-A6E7-01A-11R-A31N-07, TCGA-KK-A7B4-01A-11R-A32O-07, TCGA-CH-5744-01A-11R-1580-07, TCGA-J4-AATZ-01A-11R-A41O-07, TCGA-EJ-7781-01A-11R-2118-07, TCGA-KK-A8I9-01A-11R-A36G-07, TCGA-YL-A8SA-01A-21R-A37L-07, TCGA-HC-A6AN-01A-11R-A30B-07, TCGA-HC-8262-01A-11R-2263-07, TCGA-KK-A6E0-01A-11R-A311-07, TCGA-CH-5761-01A-11R-1580-07, TCGA-ZG-A9LB-01A-11R-A41O-07, TCGA-XK-AAK1-01A-11R-A41O-07, TCGA-YL-A8S9-01A-11R-A37L-07, TCGA-ZG-A8QY-01A-11R-A37L-07, TCGA-YL-A8SK-01B-21R-A37L-07, TCGA-ZG-A9ND-01A-11R-A41O-07, TCGA-HC-8265-01B-04R-2302-07, TCGA-WW-A8ZI-01A-11R-A37L-07, TCGA-G9-6348-01A-11R-1789-07, TCGA-EJ-5514-01A-01R-1580-07, TCGA-XQ-A8TB-01A-11R-A36G-07, TCGA-X4-A8KQ-01A-12R-A36G-07, TCGA-TK-A8OK-01A-22R-A36G-07, TCGA-KK-A8II-01A-11R-A36G-07, TCGA-G9-7521-01A-11R-2263-07, TCGA-V1-A9OQ-01A-11R-A41O-07, TCGA-HC-A631-01A-11R-A29R-07, TCGA-HC-7078-01A-11R-2118-07, TCGA-VN-A88P-01A-11R-A352-07, TCGA-XK-AAJA-01A-11R-A41O-07, TCGA-ZG-A9M4-01A-11R-A41O-07, TCGA-HC-8264-01B-11R-2403-07, TCGA-VP-A87B-01A-11R-A352-07, TCGA-ZG-A9L0-01A-11R-A41O-07, TCGA-G9-6499-01A-12R-1965-07, TCGA-V1-A9OL-01A-11R-A41O-07, TCGA-EJ-7789-01A-11R-2118-07, TCGA-ZG-A8QW-01A-11R-A37L-07, TCGA-KK-A59X-01A-11R-A29R-07, TCGA-EJ-7115-01A-11R-2118-07, TCGA-EJ-A46F-01A-31R-A250-07, TCGA-XK-AAJ3-01A-11R-A41O-07, TCGA-KK-A7AZ-01A-12R-A32O-07, TCGA-HC-7742-01A-11R-2118-07, TCGA-KC-A7FA-01A-21R-A33R-07, TCGA-VP-A875-01A-31R-A352-07, TCGA-HI-7168-01A-11R-2118-07, TCGA-HC-7738-01A-11R-2118-07, TCGA-V1-A9OH-01A-11R-A41O-07, TCGA-TP-A8TV-01A-11R-A41O-07, TCGA-J9-A52C-01A-11R-A26U-07, TCGA-EJ-5521-01A-01R-1580-07, TCGA-HC-7213-01A-11R-2118-07, TCGA-EJ-8474-01A-11R-2403-07, TCGA-XJ-A83F-01A-11R-A352-07, TCGA-XK-AAIV-01A-11R-A41O-07, TCGA-J4-A83I-01A-11R-A36G-07, TCGA-CH-5737-01A-11R-1580-07, TCGA-VP-A87K-01A-11R-A352-07, TCGA-QU-A6IO-01A-11R-A31N-07, TCGA-VP-A879-01A-11R-A352-07, TCGA-V1-A9O5-01A-11R-A41O-07, TCGA-VP-A878-01A-31R-A352-07, TCGA-KK-A8IK-01A-11R-A36G-07, TCGA-EJ-A65E-01A-11R-A29R-07, TCGA-V1-A8WL-01A-11R-A37L-07, TCGA-YL-A8HJ-01A-11R-A36G-07, TCGA-KK-A59Z-01A-12R-A26U-07, TCGA-HC-7749-01A-11R-2118-07, TCGA-QU-A6IM-01A-11R-A31N-07, TCGA-EJ-7314-01A-31R-2118-07, TCGA-H9-7775-01A-11R-2118-07, TCGA-EJ-A65J-01A-11R-A311-07, TCGA-KK-A7AP-01A-12R-A33R-07, TCGA-EJ-5518-01A-01R-1580-07, TCGA-HC-A632-01A-11R-A29R-07, TCGA-ZG-A9LZ-01A-11R-A41O-07, TCGA-EJ-5499-01A-01R-1580-07, TCGA-ZG-A9L1-01A-11R-A41O-07, TCGA-CH-5769-01A-11R-1580-07, TCGA-KK-A8I5-01A-11R-A36G-07, TCGA-HI-7171-01A-12R-2118-07, TCGA-V1-A8WV-01A-11R-A37L-07, TCGA-YL-A9WY-01A-11R-A41O-07, TCGA-EJ-5507-01A-01R-1580-07, TCGA-M7-A725-01A-12R-A32O-07, TCGA-J9-A8CP-01A-11R-A352-07, TCGA-KK-A8IB-01A-11R-A36G-07, TCGA-CH-5767-01A-11R-1789-07, TCGA-KK-A6E8-01A-11R-A31N-07, TCGA-ZG-A9L9-01A-11R-A41O-07, TCGA-ZG-A9KY-01A-11R-A41O-07, TCGA-V1-A9ZG-01A-11R-A41O-07, TCGA-2A-A8VT-01A-11R-A37L-07, TCGA-G9-6496-01A-11R-1789-07, TCGA-ZG-A8QX-01A-11R-A37L-07, TCGA-EJ-5504-01A-01R-1580-07, TCGA-EJ-7123-01A-11R-1965-07, TCGA-EJ-5496-01A-01R-1580-07, TCGA-CH-5752-01A-11R-1580-07, TCGA-EJ-5498-01A-01R-1580-07, TCGA-ZG-A9LY-01A-11R-A41O-07, TCGA-KK-A8ID-01A-11R-A36G-07, TCGA-V1-A8WW-01A-11R-A37L-07, TCGA-HC-7231-01A-11R-2118-07, TCGA-YL-A9WH-01A-11R-A37L-07, TCGA-XK-AAJR-01A-11R-A41O-07, TCGA-EJ-5524-01A-01R-1580-07, TCGA-EJ-5532-01A-01R-1580-07, TCGA-CH-5791-01A-11R-1580-07, TCGA-V1-A9OT-01A-11R-A41O-07, TCGA-EJ-5505-01A-01R-1580-07, TCGA-HC-7233-01A-11R-2118-07, TCGA-CH-5750-01A-11R-1580-07, TCGA-EJ-8472-01A-11R-2403-07, TCGA-HC-7232-01A-11R-2118-07, TCGA-V1-A8MU-01A-11R-A37L-07, TCGA-XJ-A83G-01A-11R-A352-07, TCGA-HC-8265-01A-11R-2263-07, TCGA-KC-A7F3-01A-21R-A33R-07, TCGA-YL-A8SC-01A-11R-A37L-07, TCGA-J9-A8CM-01A-11R-A352-07, TCGA-EJ-A46G-01A-31R-A26U-07, TCGA-G9-6363-01A-21R-1789-07, TCGA-KK-A8IL-01A-11R-A36G-07, TCGA-EJ-A65F-01A-21R-A311-07, TCGA-G9-6338-01A-12R-1965-07, TCGA-EJ-5525-01A-01R-1580-07, TCGA-CH-5751-01A-11R-1580-07, TCGA-CH-5753-01A-11R-1580-07, TCGA-EJ-A8FS-01A-11R-A352-07, TCGA-HC-7080-01A-11R-1965-07, TCGA-J9-A52E-01A-11R-A26U-07, TCGA-HC-A9TE-01A-11R-A41O-07, TCGA-CH-5788-01A-11R-1580-07, TCGA-TP-A8TT-01A-12R-A41O-07, TCGA-YL-A8S8-01A-11R-A37L-07, TCGA-VP-A87H-01A-11R-A352-07, TCGA-2A-A8VL-01A-21R-A37L-07, TCGA-M7-A722-01A-12R-A36G-07, TCGA-ZG-A9LN-01A-11R-A41O-07, TCGA-KK-A6E6-01A-11R-A311-07, TCGA-V1-A9ZR-01A-11R-A41O-07, TCGA-J9-A52D-01A-11R-A29R-07, TCGA-EJ-7330-01A-11R-2118-07, TCGA-EJ-7783-01A-11R-2118-07, TCGA-EJ-7317-01A-31R-2118-07, TCGA-KC-A7F5-01A-11R-A33R-07, TCGA-G9-6339-01A-12R-A311-07, TCGA-J4-A83J-01A-11R-A36G-07, TCGA-KK-A8IA-01A-11R-A36G-07, TCGA-KK-A7AW-01A-11R-A32O-07, TCGA-2A-A8W3-01A-11R-A37L-07, TCGA-EJ-7788-01A-11R-2118-07, TCGA-G9-7525-01A-31R-2263-07, TCGA-YJ-A8SW-01A-11R-A37L-07, TCGA-HC-8257-01A-11R-2263-07, TCGA-YL-A8HM-01A-11R-A36G-07, TCGA-YL-A8SL-01B-21R-A37L-07, TCGA-G9-6361-01A-21R-1965-07, TCGA-EJ-A8FN-01A-11R-A352-07, TCGA-KC-A4BV-01A-31R-A26U-07, TCGA-V1-A8MF-01A-11R-A36G-07, TCGA-KC-A7FD-01A-11R-A33R-07, TCGA-HC-7077-01A-11R-1965-07, TCGA-VN-A88Q-01A-11R-A352-07, TCGA-CH-5794-01A-11R-1580-07, TCGA-EJ-7328-01A-31R-2118-07, TCGA-YL-A8SO-01B-31R-A37L-07, TCGA-YL-A8SH-01B-11R-A37L-07, TCGA-G9-6362-01A-11R-1789-07, TCGA-CH-5772-01A-11R-1580-07, TCGA-EJ-7325-01B-11R-A32O-07, TCGA-CH-5768-01A-11R-1580-07, TCGA-XJ-A9DX-01A-11R-A37L-07, TCGA-HC-A9TH-01A-11R-A41O-07, TCGA-CH-5765-01A-11R-1580-07, TCGA-EJ-A65M-01A-11R-A29R-07, TCGA-ZG-A9N3-01A-11R-A41O-07, TCGA-CH-5792-01A-11R-1580-07, TCGA-J9-A52B-01A-11R-A26U-07, TCGA-EJ-5519-01A-01R-1580-07, TCGA-V1-A9Z7-01A-11R-A41O-07, TCGA-G9-6494-01A-11R-1789-07, TCGA-EJ-5526-01A-01R-1580-07, TCGA-XQ-A8TA-01A-11R-A36G-07, TCGA-VP-A87D-01A-11R-A352-07, TCGA-M7-A724-01A-12R-A32O-07, TCGA-V1-A9Z9-01A-21R-A41O-07, TCGA-YL-A8SQ-01B-11R-A37L-07, TCGA-KK-A59V-01A-11R-A29R-07, TCGA-KK-A7B3-01A-11R-A33R-07, TCGA-ZG-A9LM-01A-11R-A41O-07 | 486 samples with computable TMB score, consisting of 247 samples with low TMB score and 239 samples with high TMB score. |

**(4) 42 samples used for TMB analysis (Fig.6D, Fig.S1)**

| **Group** | **Dataset** | **Subset** | **Note** |
| --- | --- | --- | --- |
| **Tumor** | TCGA | TCGA-J4-A67N-01A-11D-A30E-08, TCGA-ZG-A9N3-01A-11D-A41K-08, TCGA-ZG-A9MC-01A-31D-A41K-08, TCGA-XK-AAIV-01A-11D-A41K-08, TCGA-ZG-A9KY-01A-11D-A41K-08, TCGA-G9-6498-01A-12D-A30X-08, TCGA-ZG-A9LN-01A-11D-A41K-08, TCGA-G9-7523-01A-11D-2260-08, TCGA-H9-A6BX-01A-31D-A30X-08, TCGA-EJ-A65B-01A-12D-A30E-08, TCGA-HC-A9TH-01A-11D-A41K-08, TCGA-XK-AAIW-01A-11D-A41K-08, TCGA-J9-A8CP-01A-11D-A34U-08, TCGA-ZG-A9M4-01A-11D-A41K-08, TCGA-KK-A7B0-01A-11D-A32B-08, TCGA-HC-A6AS-01A-11D-A30E-08, TCGA-CH-5751-01A-11D-1576-08, TCGA-HC-A9TE-01A-11D-A41K-08, TCGA-G9-7521-01A-11D-2260-08, TCGA-EJ-5531-01A-01D-1576-08, TCGA-ZG-A9LS-01A-12D-A41K-08, TCGA-CH-5754-01A-11D-1576-08, TCGA-V1-A8MF-01A-11D-A364-08, TCGA-M7-A723-01A-12D-A32B-08, TCGA-KK-A8I4-01A-11D-A364-08, TCGA-V1-A9O9-01A-11D-A41K-08, TCGA-YL-A9WY-01A-11D-A41K-08, TCGA-KK-A59V-01A-11D-A29Q-08, TCGA-VP-A87C-01A-11D-A34U-08, TCGA-KK-A7B2-01A-12D-A32B-08, TCGA-VN-A88I-01A-11D-A34U-08, TCGA-G9-6379-01A-11D-A31L-08, TCGA-G9-6347-01A-11D-A31L-08, TCGA-EJ-7328-01A-31D-2114-08, TCGA-KK-A7AV-01A-11D-A32B-08, TCGA-HC-8266-01A-11D-2260-08, TCGA-EJ-8470-01A-11D-2395-08, TCGA-V1-A8MU-01A-11D-A377-08, TCGA-4L-AA1F-01A-11D-A41K-08, TCGA-V1-A9O5-01A-11D-A41K-08, TCGA-J4-A67M-01A-11D-A30E-08, TCGA-XJ-A9DI-01A-11D-A377-08 | 42 samples together with TMB data and satisfy CIBERSORT p-value<0.05 |

**(5) 78 samples used for univariate survival analysis and multivariate regression analysis(Fig.8)**

| **Group** | **cohort** | **Subset** | **Note** |
| --- | --- | --- | --- |
| **Tumor** | Training cohort | TCGA-M7-A723, TCGA-ZG-A9N3, TCGA-ZG-A9MC, TCGA-ZG-A9KY, TCGA-YL-A9WY, TCGA-XK-AAIW, TCGA-VP-A87C, TCGA-VN-A88I, TCGA-V1-A9O5, TCGA-KK-A8I4, TCGA-KK-A7B2, TCGA-KK-A59V, TCGA-J9-A8CP, TCGA-J4-A6G1, TCGA-HC-A9TH, TCGA-HC-A6AS, TCGA-HC-8266, TCGA-H9-A6BX, TCGA-G9-6379, TCGA-EJ-8470, TCGA-EJ-5531, TCGA-CH-5754, GSM416520, GSM416488, GSM416480, GSM416472, GSM416428, GSM416406, GSM416398, GSM416393, GSM416382, GSM416371, GSM416317, GSM416310, GSM416302, GSM416294, GSM416269, GSM416255, GSM416253 | All 72 samples together with survival data and satisfy CIBERSORT p-value<0.05 |
|  | Validation cohort | TCGA-V1-A8MU, TCGA-ZG-A9M4, TCGA-ZG-A9LS, TCGA-ZG-A9LN, TCGA-XK-AAIV, TCGA-XJ-A9DI, TCGA-V1-A9O9, TCGA-V1-A8MF, TCGA-KK-A7B0, TCGA-KK-A7AV, TCGA-J4-A67N, TCGA-J4-A67M, TCGA-HC-A9TE, TCGA-HC-7079, TCGA-G9-7523, TCGA-G9-7521, TCGA-G9-6498, TCGA-G9-6347, TCGA-EJ-A65B, TCGA-EJ-7328, TCGA-CH-5751, TCGA-4L-AA1F, GSM416512, GSM416511, GSM416504, GSM416493, GSM416483, GSM416471, GSM416458, GSM416439, GSM416427, GSM416420, GSM416405, GSM416395, GSM416380, GSM416364, GSM416266, GSM416248, GSM416246 |  |

**(6) 551 samples used for differential genes analysis(Fig.S2A,B)**

| **Group** | **Dataset** | **Subset** | **Note** |
| --- | --- | --- | --- |
| **Normal** | TCGA | TCGA-G9-6496-11A-01R-1789-07, TCGA-G9-6363-11A-01R-1789-07, TCGA-HC-7211-11A-01R-2118-07, TCGA-CH-5761-11A-01R-1580-07, TCGA-EJ-7115-11A-01R-2118-07, TCGA-G9-6333-11A-01R-1965-07, TCGA-HC-8262-11A-01R-2263-07, TCGA-HC-8260-11A-01R-2263-07, TCGA-HC-7740-11A-01R-2118-07, TCGA-EJ-7331-11A-01R-2118-07, TCGA-EJ-7794-11A-01R-2118-07, TCGA-EJ-7314-11A-01R-2118-07, TCGA-EJ-7786-11A-01R-2118-07, TCGA-EJ-7792-11A-01R-2118-07, TCGA-HC-7745-11A-01R-2118-07, TCGA-EJ-7321-11A-01R-2263-07, TCGA-HC-7738-11A-01R-2118-07, TCGA-EJ-7125-11A-01R-1965-07, TCGA-HC-8258-11A-01R-2263-07, TCGA-EJ-7317-11A-01R-2118-07, TCGA-G9-6342-11A-02R-1965-07, TCGA-EJ-7783-11A-01R-2118-07, TCGA-EJ-7793-11A-01R-2263-07, TCGA-EJ-7781-11A-01R-2118-07, TCGA-HC-7752-11A-01R-2118-07, TCGA-G9-6351-11A-01R-1965-07, TCGA-EJ-A8FO-11A-11R-A36G-07, TCGA-HC-7819-11A-01R-2118-07, TCGA-HC-7747-11A-01R-2118-07, TCGA-EJ-7123-11A-01R-1965-07, TCGA-G9-6362-11A-01R-1789-07, TCGA-EJ-7784-11A-01R-2118-07, TCGA-J4-A83J-11A-11R-A36G-07, TCGA-G9-6499-11A-02R-1965-07, TCGA-EJ-7782-11A-01R-2118-07, TCGA-EJ-7330-11A-01R-2118-07, TCGA-G9-6348-11A-01R-1789-07, TCGA-G9-6356-11A-01R-1789-07, TCGA-EJ-7785-11A-01R-2118-07, TCGA-G9-6384-11A-01R-1858-07, TCGA-EJ-7328-11A-01R-2118-07, TCGA-EJ-7315-11A-01R-2118-07, TCGA-CH-5767-11B-01R-1789-07, TCGA-EJ-7797-11A-01R-2263-07, TCGA-CH-5768-11A-01R-1580-07, TCGA-EJ-7789-11A-01R-2118-07, TCGA-HC-8259-11A-01R-2263-07, TCGA-HC-7737-11A-02R-2118-07, TCGA-EJ-7327-11A-01R-2118-07, TCGA-CH-5769-11A-01R-1580-07, TCGA-HC-7742-11A-01R-2118-07, TCGA-G9-6365-11A-01R-1789-07, TCGA-2A-A8VO-01A-11R-A37L-07 |  |
| **Tumor** | TCGA | TCGA-EJ-A65G-01A-21R-A29R-07, TCGA-YL-A8SR-01B-11R-A37L-07, TCGA-QU-A6IL-01A-11R-A31N-07, TCGA-ZG-A9LS-01A-12R-A41O-07, TCGA-G9-6329-01A-13R-1965-07, TCGA-KK-A7AY-01A-11R-A33R-07, TCGA-CH-5762-01A-11R-1580-07, TCGA-KK-A7AU-01A-11R-A32O-07, TCGA-YL-A9WI-01A-11R-A37L-07, TCGA-XK-AAJT-01A-11R-A41O-07, TCGA-EJ-A6RC-01A-11R-A32O-07, TCGA-EJ-7784-01A-11R-2118-07, TCGA-FC-7961-01A-11R-A29R-07, TCGA-EJ-A7NN-01A-11R-A33R-07, TCGA-EJ-5506-01A-01R-1580-07, TCGA-EJ-7218-01B-11R-A32O-07, TCGA-Y6-A8TL-01A-21R-A37L-07, TCGA-VN-A88K-01A-11R-A352-07, TCGA-EJ-A65D-01A-11R-A30B-07, TCGA-HC-7748-01A-11R-2118-07, TCGA-2A-AAYU-01A-11R-A41O-07, TCGA-2A-A8W1-01A-11R-A37L-07, TCGA-FC-A4JI-01A-11R-A250-07, TCGA-VN-A88O-01A-11R-A352-07, TCGA-HC-A6HY-01A-11R-A31N-07, TCGA-KC-A4BR-01A-32R-A32Y-07, TCGA-YL-A9WJ-01A-11R-A37L-07, TCGA-VN-A88N-01A-11R-A36G-07, TCGA-HC-8261-01A-11R-2263-07, TCGA-J4-A67M-01A-11R-A30B-07, TCGA-FC-A6HD-01A-11R-A31N-07, TCGA-HC-A6AO-01A-11R-A30B-07, TCGA-CH-5739-01A-11R-1580-07, TCGA-G9-6379-01A-11R-A31N-07, TCGA-G9-6343-01A-21R-1965-07, TCGA-HC-8216-01A-11R-A29R-07, TCGA-YL-A8SB-01A-31R-A37L-07, TCGA-J4-AAU2-01A-11R-A41O-07, TCGA-J9-A8CL-01A-11R-A352-07, TCGA-KK-A8I4-01A-11R-A36G-07, TCGA-V1-A9OA-01A-11R-A41O-07, TCGA-KK-A8IF-01A-11R-A36G-07, TCGA-KK-A7B1-01A-11R-A32O-07, TCGA-VP-AA1N-01A-31R-A41O-07, TCGA-XK-AAIW-01A-11R-A41O-07, TCGA-ZG-A9L5-01A-12R-A41O-07, TCGA-G9-7522-01A-11R-2263-07, TCGA-J4-A67N-01A-11R-A30B-07, TCGA-HC-A6AL-01A-11R-A30B-07, TCGA-EJ-8469-01A-11R-2403-07, TCGA-EJ-A46D-01A-21R-A32Y-07, TCGA-G9-6367-01A-11R-1789-07, TCGA-VP-A87E-01A-31R-A352-07, TCGA-HC-A4ZV-01A-11R-A26U-07, TCGA-G9-6373-01A-11R-1789-07, TCGA-HC-8258-01B-05R-2302-07, TCGA-G9-6370-01A-11R-1789-07, TCGA-VP-A87J-01A-11R-A352-07, TCGA-CH-5763-01A-11R-1580-07, TCGA-ZG-A9NI-01A-11R-A41O-07, TCGA-KK-A6E4-01A-11R-A30B-07, TCGA-EJ-A7NF-01A-11R-A33R-07, TCGA-KC-A4BN-01A-61R-A250-07, TCGA-J4-A83M-01A-11R-A352-07, TCGA-EJ-5517-01A-01R-1580-07, TCGA-CH-5754-01A-11R-1580-07, TCGA-EJ-A46H-01A-31R-A26U-07, TCGA-2A-AAYF-01A-11R-A41O-07, TCGA-YL-A8HK-01A-11R-A36G-07, TCGA-2A-A8VX-01A-11R-A37L-07, TCGA-HC-A48F-01A-11R-A250-07, TCGA-FC-A66V-01A-21R-A30B-07, TCGA-HC-7736-01A-11R-2118-07, TCGA-V1-A9O9-01A-11R-A41O-07, TCGA-CH-5789-01A-11R-1580-07, TCGA-HC-A8D1-01A-11R-A36G-07, TCGA-EJ-8468-01A-21R-2403-07, TCGA-V1-A9O5-06A-11R-A41O-07, TCGA-V1-A9ZK-01A-11R-A41O-07, TCGA-KC-A7FE-01A-12R-A33R-07, TCGA-Y6-A9XI-01A-11R-A41O-07, TCGA-HC-7210-01A-11R-2118-07, TCGA-G9-6336-01A-11R-1789-07, TCGA-KK-A6E2-01A-11R-A311-07, TCGA-KK-A5A1-01A-11R-A29R-07, TCGA-EJ-A7NK-01A-12R-A352-07, TCGA-2A-AAYO-01A-11R-A41O-07, TCGA-V1-A9ZI-01A-11R-A41O-07, TCGA-G9-A9S4-01A-11R-A41O-07, TCGA-VP-A872-01A-11R-A352-07, TCGA-KK-A7B0-01A-11R-A32O-07, TCGA-J4-8198-01A-11R-2263-07, TCGA-HC-7819-01A-11R-2118-07, TCGA-HC-8259-01A-11R-2263-07, TCGA-EJ-5511-01A-01R-1580-07, TCGA-EJ-5495-01A-01R-1580-07, TCGA-ZG-A9L2-01A-31R-A41O-07, TCGA-EJ-A7NJ-01A-22R-A352-07, TCGA-G9-6353-01A-11R-1965-07, TCGA-HC-7821-01A-12R-2118-07, TCGA-V1-A9OX-01A-11R-A41O-07, TCGA-EJ-7782-01A-11R-2118-07, TCGA-V1-A8MG-01A-11R-A36G-07, TCGA-XK-AAJP-01A-11R-A41O-07, TCGA-EJ-A46I-01A-12R-A26U-07, TCGA-G9-A9S7-01A-11R-A41O-07, TCGA-HC-A76W-01A-11R-A33R-07, TCGA-EJ-AB20-01A-12R-A41O-07, TCGA-EJ-A7NM-01A-21R-A33R-07, TCGA-V1-A8X3-01A-11R-A37L-07, TCGA-HC-A8CY-01A-11R-A36G-07, TCGA-VN-A88I-01A-11R-A352-07, TCGA-HC-7740-01A-11R-2118-07, TCGA-KK-A6E7-01A-11R-A31N-07, TCGA-KK-A7B4-01A-11R-A32O-07, TCGA-YL-A9WX-01A-21R-A41O-07, TCGA-CH-5744-01A-11R-1580-07, TCGA-J4-AATZ-01A-11R-A41O-07, TCGA-HC-8266-01A-11R-2263-07, TCGA-EJ-7781-01A-11R-2118-07, TCGA-KK-A8I9-01A-11R-A36G-07, TCGA-YL-A8SA-01A-21R-A37L-07, TCGA-HC-A6AN-01A-11R-A30B-07, TCGA-HC-8262-01A-11R-2263-07, TCGA-EJ-A46B-01A-31R-A250-07, TCGA-KK-A59Y-01A-11R-A26U-07, TCGA-EJ-8470-01A-11R-2403-07, TCGA-XK-AAIR-01A-11R-A41O-07, TCGA-KK-A6E0-01A-11R-A311-07, TCGA-HC-7740-01B-04R-2302-07, TCGA-G9-7509-01A-11R-A41O-07, TCGA-HC-7817-01B-11R-A29R-07, TCGA-CH-5761-01A-11R-1580-07, TCGA-ZG-A8QZ-01A-11R-A37L-07, TCGA-G9-6356-01A-11R-1789-07, TCGA-G9-6333-01A-12R-1965-07, TCGA-EJ-7794-01A-11R-2118-07, TCGA-ZG-A9LB-01A-11R-A41O-07, TCGA-XK-AAK1-01A-11R-A41O-07, TCGA-YL-A8S9-01A-11R-A37L-07, TCGA-J4-8200-01A-11R-A29R-07, TCGA-ZG-A9L4-01A-11R-A41O-07, TCGA-ZG-A8QY-01A-11R-A37L-07, TCGA-YL-A8SK-01B-21R-A37L-07, TCGA-ZG-A9ND-01A-11R-A41O-07, TCGA-KK-A6E1-01A-11R-A311-07, TCGA-EJ-5522-01A-01R-1580-07, TCGA-HC-8265-01B-04R-2302-07, TCGA-WW-A8ZI-01A-11R-A37L-07, TCGA-G9-6348-01A-11R-1789-07, TCGA-VP-A87C-01A-11R-A352-07, TCGA-EJ-5514-01A-01R-1580-07, TCGA-EJ-7786-01A-11R-2118-07, TCGA-XQ-A8TB-01A-11R-A36G-07, TCGA-CH-5766-01A-11R-1580-07, TCGA-X4-A8KQ-01A-12R-A36G-07, TCGA-J9-A8CN-01A-11R-A352-07, TCGA-YL-A8SJ-01B-11R-A37L-07, TCGA-HC-A8D0-01A-11R-A36G-07, TCGA-TK-A8OK-01A-22R-A36G-07, TCGA-KK-A8II-01A-11R-A36G-07, TCGA-EJ-A7NG-01A-31R-A33R-07, TCGA-HC-8260-01A-11R-2263-07, TCGA-KK-A8I7-01A-21R-A36G-07, TCGA-EJ-5510-01A-01R-1580-07, TCGA-KK-A7B2-01A-12R-A32O-07, TCGA-EJ-A46E-01A-31R-A250-07, TCGA-G9-7521-01A-11R-2263-07, TCGA-V1-A9OQ-01A-11R-A41O-07, TCGA-G9-6364-01A-21R-1789-07, TCGA-HC-A631-01A-11R-A29R-07, TCGA-G9-6366-01A-11R-2118-07, TCGA-HC-7078-01A-11R-2118-07, TCGA-VN-A88P-01A-11R-A352-07, TCGA-XK-AAJA-01A-11R-A41O-07, TCGA-ZG-A9M4-01A-11R-A41O-07, TCGA-HC-8264-01B-11R-2403-07, TCGA-KK-A8IG-01A-11R-A36G-07, TCGA-VP-A87B-01A-11R-A352-07, TCGA-V1-A8ML-01A-11R-A37L-07, TCGA-G9-6385-01A-11R-1789-07, TCGA-G9-6347-01A-11R-A31N-07, TCGA-J4-A67T-01A-11R-A311-07, TCGA-ZG-A9L0-01A-11R-A41O-07, TCGA-G9-6499-01A-12R-1965-07, TCGA-V1-A9OL-01A-11R-A41O-07, TCGA-EJ-7789-01A-11R-2118-07, TCGA-ZG-A8QW-01A-11R-A37L-07, TCGA-KK-A59X-01A-11R-A29R-07, TCGA-M7-A721-01A-12R-A32O-07, TCGA-EJ-7115-01A-11R-2118-07, TCGA-EJ-A46F-01A-31R-A250-07, TCGA-M7-A723-01A-12R-A32O-07, TCGA-XK-AAJ3-01A-11R-A41O-07, TCGA-KK-A7AZ-01A-12R-A32O-07, TCGA-HC-7742-01A-11R-2118-07, TCGA-HC-7745-01A-11R-2118-07, TCGA-V1-A8MM-01A-11R-A37L-07, TCGA-KC-A7FA-01A-21R-A33R-07, TCGA-VP-A875-01A-31R-A352-07, TCGA-HC-7081-01A-11R-1965-07, TCGA-CH-5745-01A-11R-1580-07, TCGA-HI-7168-01A-11R-2118-07, TCGA-HC-7738-01A-11R-2118-07, TCGA-V1-A9OH-01A-11R-A41O-07, TCGA-TP-A8TV-01A-11R-A41O-07, TCGA-J9-A52C-01A-11R-A26U-07, TCGA-YL-A9WL-01A-11R-A41O-07, TCGA-EJ-5521-01A-01R-1580-07, TCGA-EJ-5516-01A-01R-1580-07, TCGA-CH-5738-01A-11R-1580-07, TCGA-G9-7519-01A-11R-2263-07, TCGA-HC-7213-01A-11R-2118-07, TCGA-G9-6384-01A-11R-1789-07, TCGA-EJ-8474-01A-11R-2403-07, TCGA-HC-A6AQ-01A-11R-A30B-07, TCGA-EJ-7793-01A-31R-2263-07, TCGA-HC-7752-01A-11R-2118-07, TCGA-XJ-A83F-01A-11R-A352-07, TCGA-EJ-5508-01A-02R-1580-07, TCGA-KK-A7AQ-01A-11R-A33R-07, TCGA-XK-AAIV-01A-11R-A41O-07, TCGA-EJ-7315-01A-31R-2118-07, TCGA-J4-A83I-01A-11R-A36G-07, TCGA-EJ-7312-01B-21R-A32O-07, TCGA-EJ-A7NH-01A-12R-A33R-07, TCGA-CH-5737-01A-11R-1580-07, TCGA-EJ-A65B-01A-12R-A30B-07, TCGA-CH-5764-01A-21R-1580-07, TCGA-VN-A88M-01A-11R-A352-07, TCGA-EJ-5503-01A-01R-1580-07, TCGA-J4-A67L-01A-11R-A30B-07, TCGA-EJ-5530-01A-01R-1580-07, TCGA-VP-A87K-01A-11R-A352-07, TCGA-QU-A6IO-01A-11R-A31N-07, TCGA-VP-A879-01A-11R-A352-07, TCGA-V1-A9O5-01A-11R-A41O-07, TCGA-VP-A878-01A-31R-A352-07, TCGA-4L-AA1F-01A-11R-A41O-07, TCGA-KC-A7F6-01A-11R-A33R-07, TCGA-KK-A8IK-01A-11R-A36G-07, TCGA-EJ-A65E-01A-11R-A29R-07, TCGA-EJ-7792-01A-11R-2118-07, TCGA-KK-A8I8-01A-11R-A36G-07, TCGA-V1-A8WL-01A-11R-A37L-07, TCGA-YL-A8HJ-01A-11R-A36G-07, TCGA-KK-A59Z-01A-12R-A26U-07, TCGA-HC-7749-01A-11R-2118-07, TCGA-HC-A6AP-01A-11R-A30B-07, TCGA-QU-A6IM-01A-11R-A31N-07, TCGA-EJ-7314-01A-31R-2118-07, TCGA-J4-AATV-01A-11R-A41O-07, TCGA-H9-7775-01A-11R-2118-07, TCGA-EJ-A65J-01A-11R-A311-07, TCGA-KK-A7AP-01A-12R-A33R-07, TCGA-YL-A8HL-01A-11R-A36G-07, TCGA-EJ-5518-01A-01R-1580-07, TCGA-HC-A632-01A-11R-A29R-07, TCGA-ZG-A9LZ-01A-11R-A41O-07, TCGA-EJ-5499-01A-01R-1580-07, TCGA-ZG-A9L1-01A-11R-A41O-07, TCGA-CH-5769-01A-11R-1580-07, TCGA-G9-6378-01A-11R-1789-07, TCGA-FC-7708-01A-11R-2118-07, TCGA-KK-A6E3-01A-21R-A30B-07, TCGA-M7-A720-01A-12R-A32O-07, TCGA-KK-A8I5-01A-11R-A36G-07, TCGA-HI-7171-01A-12R-2118-07, TCGA-G9-6342-01A-11R-1965-07, TCGA-V1-A8WV-01A-11R-A37L-07, TCGA-YL-A9WY-01A-11R-A41O-07, TCGA-EJ-5507-01A-01R-1580-07, TCGA-M7-A725-01A-12R-A32O-07, TCGA-HC-7230-01A-11R-2118-07, TCGA-J4-A67R-01A-21R-A30B-07, TCGA-J9-A8CP-01A-11R-A352-07, TCGA-KK-A8IB-01A-11R-A36G-07, TCGA-CH-5767-01A-11R-1789-07, TCGA-EJ-5512-01A-01R-1580-07, TCGA-KK-A6E8-01A-11R-A31N-07, TCGA-M7-A71Z-01A-12R-A32O-07, TCGA-ZG-A9L9-01A-11R-A41O-07, TCGA-G9-A9S0-01A-11R-A41O-07, TCGA-ZG-A9KY-01A-11R-A41O-07, TCGA-V1-A9ZG-01A-11R-A41O-07, TCGA-2A-A8VT-01A-11R-A37L-07, TCGA-G9-6496-01A-11R-1789-07, TCGA-ZG-A8QX-01A-11R-A37L-07, TCGA-EJ-5504-01A-01R-1580-07, TCGA-EJ-7123-01A-11R-1965-07, TCGA-HC-8258-01A-11R-2263-07, TCGA-EJ-A8FO-01A-21R-A36G-07, TCGA-HC-7744-01A-11R-2118-07, TCGA-M7-A71Y-01A-22R-A32O-07, TCGA-EJ-A6RA-01A-11R-A33R-07, TCGA-EJ-5496-01A-01R-1580-07, TCGA-CH-5752-01A-11R-1580-07, TCGA-CH-5741-01A-11R-1580-07, TCGA-EJ-5498-01A-01R-1580-07, TCGA-ZG-A9LY-01A-11R-A41O-07, TCGA-YL-A9WK-01A-11R-A37L-07, TCGA-KK-A8ID-01A-11R-A36G-07, TCGA-G9-6369-01A-21R-1965-07, TCGA-HC-7820-01A-11R-2118-07, TCGA-G9-6498-01A-12R-A311-07, TCGA-HC-7075-01A-11R-1965-07, TCGA-V1-A8WW-01A-11R-A37L-07, TCGA-ZG-A9L6-01A-11R-A41O-07, TCGA-HC-8213-01A-11R-A29R-07, TCGA-HC-7231-01A-11R-2118-07, TCGA-YL-A9WH-01A-11R-A37L-07, TCGA-XK-AAJR-01A-11R-A41O-07, TCGA-EJ-5524-01A-01R-1580-07, TCGA-EJ-5532-01A-01R-1580-07, TCGA-CH-5791-01A-11R-1580-07, TCGA-V1-A9OT-01A-11R-A41O-07, TCGA-CH-5740-01A-11R-1580-07, TCGA-G9-7523-01A-11R-2263-07, TCGA-CH-5771-01A-21R-1580-07, TCGA-EJ-5505-01A-01R-1580-07, TCGA-HC-7233-01A-11R-2118-07, TCGA-CH-5750-01A-11R-1580-07, TCGA-HC-7818-01A-11R-2118-07, TCGA-EJ-8472-01A-11R-2403-07, TCGA-HC-7232-01A-11R-2118-07, TCGA-V1-A8MU-01A-11R-A37L-07, TCGA-XJ-A83G-01A-11R-A352-07, TCGA-HC-8265-01A-11R-2263-07, TCGA-KC-A7F3-01A-21R-A33R-07, TCGA-YL-A8SI-01A-11R-A41O-07, TCGA-YL-A8SC-01A-11R-A37L-07, TCGA-G9-6365-01A-11R-1789-07, TCGA-H9-A6BY-01A-11R-A30B-07, TCGA-J4-A67S-01A-11R-A30B-07, TCGA-J9-A8CM-01A-11R-A352-07, TCGA-G9-6332-01A-11R-1789-07, TCGA-HC-7079-01A-11R-1965-07, TCGA-EJ-5531-01A-01R-1580-07, TCGA-EJ-7785-01A-11R-2118-07, TCGA-EJ-A46G-01A-31R-A26U-07, TCGA-G9-6363-01A-21R-1789-07, TCGA-H9-A6BX-01A-31R-A311-07, TCGA-V1-A8WN-01A-11R-A37L-07, TCGA-KK-A8IL-01A-11R-A36G-07, TCGA-EJ-A65F-01A-21R-A311-07, TCGA-G9-6338-01A-12R-1965-07, TCGA-EJ-A8FP-01A-21R-A36G-07, TCGA-V1-A8MK-01A-11R-A36G-07, TCGA-EJ-7331-01A-11R-2118-07, TCGA-G9-6377-01A-11R-1965-07, TCGA-EJ-5525-01A-01R-1580-07, TCGA-CH-5751-01A-11R-1580-07, TCGA-CH-5753-01A-11R-1580-07, TCGA-EJ-A8FS-01A-11R-A352-07, TCGA-HC-7080-01A-11R-1965-07, TCGA-J9-A52E-01A-11R-A26U-07, TCGA-HC-A9TE-01A-11R-A41O-07, TCGA-X4-A8KS-01A-12R-A36G-07, TCGA-CH-5788-01A-11R-1580-07, TCGA-G9-7510-01A-11R-2263-07, TCGA-TP-A8TT-01A-12R-A41O-07, TCGA-HC-8256-01A-11R-2263-07, TCGA-VP-A876-01A-11R-A352-07, TCGA-YL-A8S8-01A-11R-A37L-07, TCGA-VP-A87H-01A-11R-A352-07, TCGA-2A-A8VL-01A-21R-A37L-07, TCGA-KK-A8I6-01A-11R-A36G-07, TCGA-M7-A722-01A-12R-A36G-07, TCGA-2A-A8VV-01A-11R-A37L-07, TCGA-G9-6371-01A-11R-1789-07, TCGA-J4-A67K-01A-21R-A30B-07, TCGA-EJ-7125-01A-11R-1965-07, TCGA-V1-A9OY-01A-11R-A41O-07, TCGA-ZG-A9LN-01A-11R-A41O-07, TCGA-KK-A6E6-01A-11R-A311-07, TCGA-CH-5748-01A-11R-1580-07, TCGA-CH-5743-01A-21R-1580-07, TCGA-KK-A8IC-01A-11R-A36G-07, TCGA-EJ-A8FU-01A-11R-A36G-07, TCGA-V1-A9ZR-01A-11R-A41O-07, TCGA-J9-A52D-01A-11R-A29R-07, TCGA-XJ-A83H-01A-11R-A352-07, TCGA-EJ-7330-01A-11R-2118-07, TCGA-J4-A83K-01A-11R-A352-07, TCGA-EJ-7783-01A-11R-2118-07, TCGA-EJ-7317-01A-31R-2118-07, TCGA-XJ-A9DI-01A-11R-A37L-07, TCGA-J4-A67O-01A-11R-A30B-07, TCGA-KC-A7F5-01A-11R-A33R-07, TCGA-J4-A6M7-01A-11R-A31N-07, TCGA-G9-6339-01A-12R-A311-07, TCGA-XK-AAJU-01A-11R-A41O-07, TCGA-EJ-5515-01A-01R-1580-07, TCGA-J4-A6G3-01A-11R-A311-07, TCGA-SU-A7E7-01A-22R-A33R-07, TCGA-CH-5746-01A-11R-1580-07, TCGA-HC-7209-01A-11R-2118-07, TCGA-J4-A83J-01A-11R-A36G-07, TCGA-KK-A8IA-01A-11R-A36G-07, TCGA-EJ-7797-01A-11R-2263-07, TCGA-KK-A7AW-01A-11R-A32O-07, TCGA-2A-A8W3-01A-11R-A37L-07, TCGA-EJ-7788-01A-11R-2118-07, TCGA-G9-7525-01A-31R-2263-07, TCGA-YJ-A8SW-01A-11R-A37L-07, TCGA-KK-A6DY-01A-12R-A311-07, TCGA-HC-8257-01A-11R-2263-07, TCGA-EJ-7321-01A-31R-2263-07, TCGA-KK-A8IH-01A-11R-A36G-07, TCGA-HI-7169-01A-11R-2118-07, TCGA-YL-A8HM-01A-11R-A36G-07, TCGA-EJ-7318-01B-11R-A32O-07, TCGA-MG-AAMC-01A-11R-A41O-07, TCGA-YL-A8SL-01B-21R-A37L-07, TCGA-G9-6361-01A-21R-1965-07, TCGA-KK-A8IM-01A-11R-A36G-07, TCGA-EJ-A8FN-01A-11R-A352-07, TCGA-YL-A8SP-01B-11R-A37L-07, TCGA-KC-A4BV-01A-31R-A26U-07, TCGA-V1-A8MF-01A-11R-A36G-07, TCGA-XJ-A9DQ-01A-11R-A37L-07, TCGA-EJ-7327-01A-11R-2118-07, TCGA-KC-A7FD-01A-11R-A33R-07, TCGA-HC-7077-01A-11R-1965-07, TCGA-G9-6354-01A-11R-A311-07, TCGA-V1-A8WS-01A-11R-A37L-07, TCGA-J4-A67Q-01A-21R-A30B-07, TCGA-VN-A88Q-01A-11R-A352-07, TCGA-CH-5794-01A-11R-1580-07, TCGA-J4-A83L-01A-11R-A352-07, TCGA-G9-6351-01A-21R-1965-07, TCGA-J9-A8CK-01A-11R-A352-07, TCGA-EJ-7791-01A-11R-2118-07, TCGA-EJ-7328-01A-31R-2118-07, TCGA-HC-A6HX-01A-11R-A31N-07, TCGA-YL-A8SO-01B-31R-A37L-07, TCGA-KC-A4BL-01A-31R-A250-07, TCGA-EJ-5497-01A-02R-1580-07, TCGA-YL-A8SH-01B-11R-A37L-07, TCGA-HC-A76X-01A-11R-A33R-07, TCGA-G9-6362-01A-11R-1789-07, TCGA-CH-5772-01A-11R-1580-07, TCGA-FC-A5OB-01A-11R-A29R-07, TCGA-QU-A6IP-01A-11R-A31N-07, TCGA-VN-A88R-01A-11R-A36G-07, TCGA-J4-A6G1-01A-11R-A311-07, TCGA-QU-A6IN-01A-11R-A31N-07, TCGA-EJ-7325-01B-11R-A32O-07, TCGA-VN-A88L-01A-11R-A352-07, TCGA-EJ-5501-01A-01R-1580-07, TCGA-CH-5768-01A-11R-1580-07, TCGA-EJ-5494-01A-01R-1580-07, TCGA-HC-7737-01A-11R-2118-07, TCGA-KK-A8IJ-01A-11R-A352-07, TCGA-XJ-A9DX-01A-11R-A37L-07, TCGA-HC-A9TH-01A-11R-A41O-07, TCGA-CH-5765-01A-11R-1580-07, TCGA-KK-A6E5-01A-11R-A311-07, TCGA-EJ-5502-01A-01R-1580-07, TCGA-V1-A9Z8-01A-11R-A41O-07, TCGA-KK-A7AV-01A-11R-A32O-07, TCGA-EJ-A65M-01A-11R-A29R-07, TCGA-FC-A8O0-01A-41R-A37L-07, TCGA-YL-A8HO-01A-11R-A36G-07, TCGA-ZG-A9N3-01A-11R-A41O-07, TCGA-ZG-A9MC-01A-31R-A41O-07, TCGA-CH-5792-01A-11R-1580-07, TCGA-J9-A52B-01A-11R-A26U-07, TCGA-EJ-5519-01A-01R-1580-07, TCGA-XJ-A9DK-01A-11R-A37L-07, TCGA-V1-A9Z7-01A-11R-A41O-07, TCGA-HC-7750-01A-11R-2118-07, TCGA-HI-7170-01A-11R-2118-07, TCGA-G9-6494-01A-11R-1789-07, TCGA-HC-A6AS-01A-11R-A30B-07, TCGA-EJ-5542-01A-01R-1580-07, TCGA-EJ-5526-01A-01R-1580-07, TCGA-XQ-A8TA-01A-11R-A36G-07, TCGA-VP-A87D-01A-11R-A352-07, TCGA-M7-A724-01A-12R-A32O-07, TCGA-HC-7212-01A-11R-2118-07, TCGA-J4-A83N-01A-11R-A352-07, TCGA-CH-5790-01A-11R-1580-07, TCGA-EJ-AB27-01A-11R-A41O-07, TCGA-XA-A8JR-01A-11R-A36G-07, TCGA-EJ-5509-01A-01R-1580-07, TCGA-VN-A943-01A-11R-A41O-07, TCGA-V1-A9Z9-01A-21R-A41O-07, TCGA-HC-7211-01A-11R-2118-07, TCGA-V1-A9O7-01A-21R-A41O-07, TCGA-YL-A8SQ-01B-11R-A37L-07, TCGA-KK-A59V-01A-11R-A29R-07, TCGA-V1-A9OF-01A-11R-A41O-07, TCGA-KK-A7B3-01A-11R-A33R-07, TCGA-EJ-5527-01A-01R-1580-07, TCGA-ZG-A9LM-01A-11R-A41O-07, TCGA-HC-7747-01A-11R-2118-07, TCGA-ZG-A9LU-01A-11R-A41O-07 |  |

**(7) 495 samples used for univariate survival analysis and multivariate regression analysis(Fig.S2C)**

| **Group** | **Dataset** | **Subset** | **Note** |
| --- | --- | --- | --- |
| **Tumor** | TCGA | TCGA-VP-A875, TCGA-VP-A87J, TCGA-CH-5746, TCGA-2A-A8VX, TCGA-EJ-5504, TCGA-FC-A66V, TCGA-V1-A8MM, TCGA-CH-5744, TCGA-EJ-5542, TCGA-YL-A8SI, TCGA-EJ-A46E, TCGA-V1-A9ZR, TCGA-KC-A7FA, TCGA-ZG-A9L0, TCGA-KC-A7F3, TCGA-V1-A9ZK, TCGA-CH-5767, TCGA-KK-A6DY, TCGA-YJ-A8SW, TCGA-EJ-A7NG, TCGA-CH-5788, TCGA-EJ-A7NK, TCGA-EJ-7781, TCGA-EJ-5524, TCGA-YL-A9WH, TCGA-HC-7232, TCGA-YL-A9WK, TCGA-G9-6498, TCGA-KK-A7AP, TCGA-V1-A9Z8, TCGA-ZG-A9LB, TCGA-J4-A83N, TCGA-HC-A6AN, TCGA-EJ-AB27, TCGA-EJ-7318, TCGA-KK-A6E6, TCGA-G9-6329, TCGA-HC-A8CY, TCGA-XQ-A8TA, TCGA-J4-A67O, TCGA-EJ-A8FS, TCGA-ZG-A9LM, TCGA-EJ-5510, TCGA-G9-6354, TCGA-KK-A8I7, TCGA-XJ-A83F, TCGA-VN-A88L, TCGA-V1-A9O9, TCGA-KC-A4BL, TCGA-KK-A5A1, TCGA-V1-A9OF, TCGA-HC-A76W, TCGA-G9-6339, TCGA-EJ-7125, TCGA-HC-8259, TCGA-MG-AAMC, TCGA-V1-A8WV, TCGA-HC-7209, TCGA-G9-6333, TCGA-EJ-5532, TCGA-ZG-A9L6, TCGA-G9-6363, TCGA-HC-A6AS, TCGA-2A-A8VL, TCGA-YL-A9WI, TCGA-V1-A9O7, TCGA-XK-AAIW, TCGA-KK-A8II, TCGA-HC-8262, TCGA-EJ-7785, TCGA-FC-A5OB, TCGA-HC-8256, TCGA-V1-A9O5, TCGA-XA-A8JR, TCGA-V1-A8MU, TCGA-V1-A9ZG, TCGA-ZG-A9N3, TCGA-EJ-A65B, TCGA-CH-5764, TCGA-J4-A6G3, TCGA-G9-7523, TCGA-QU-A6IO, TCGA-ZG-A9NI, TCGA-EJ-8474, TCGA-V1-A9OT, TCGA-HC-A9TH, TCGA-V1-A8X3, TCGA-KK-A7B2, TCGA-HC-7233, TCGA-J9-A8CP, TCGA-G9-6347, TCGA-J4-8200, TCGA-KK-A7B1, TCGA-G9-6336, TCGA-EJ-7115, TCGA-ZG-A8QY, TCGA-HC-8258, TCGA-XJ-A9DI, TCGA-V1-A9OL, TCGA-EJ-7783, TCGA-KK-A59V, TCGA-HC-8257, TCGA-G9-6496, TCGA-EJ-5505, TCGA-J9-A8CL, TCGA-EJ-7328, TCGA-EJ-5501, TCGA-V1-A8MK, TCGA-VP-A87E, TCGA-J4-A67K, TCGA-KC-A4BV, TCGA-G9-6378, TCGA-HI-7168, TCGA-CH-5771, TCGA-V1-A9OX, TCGA-KK-A59Y, TCGA-V1-A8WL, TCGA-EJ-8469, TCGA-M7-A71Y, TCGA-J4-A6G1, TCGA-EJ-5494, TCGA-J9-A8CN, TCGA-EJ-5526, TCGA-EJ-5530, TCGA-EJ-A46F, TCGA-G9-6499, TCGA-J9-A52C, TCGA-HI-7171, TCGA-HC-7075, TCGA-EJ-7789, TCGA-HC-7748, TCGA-EJ-7218, TCGA-Y6-A9XI, TCGA-V1-A8MG, TCGA-2A-A8W1, TCGA-YL-A8SR, TCGA-CH-5752, TCGA-EJ-A7NH, TCGA-VN-A88R, TCGA-V1-A9OA, TCGA-KK-A8I8, TCGA-HI-7170, TCGA-HC-7210, TCGA-HC-A6HY, TCGA-EJ-7123, TCGA-KK-A8IL, TCGA-HC-A48F, TCGA-ZG-A9L4, TCGA-H9-7775, TCGA-XK-AAJT, TCGA-EJ-5502, TCGA-HC-7820, TCGA-EJ-5503, TCGA-2A-A8VT, TCGA-4L-AA1F, TCGA-KC-A7FD, TCGA-HC-7736, TCGA-V1-A8ML, TCGA-G9-6348, TCGA-VN-A88Q, TCGA-EJ-A46G, TCGA-YL-A8HM, TCGA-G9-7510, TCGA-HC-A6AP, TCGA-G9-6367, TCGA-EJ-5508, TCGA-2A-A8W3, TCGA-HC-7818, TCGA-V1-A9Z7, TCGA-HC-7211, TCGA-XJ-A9DQ, TCGA-G9-7522, TCGA-YL-A8HL, TCGA-EJ-A8FU, TCGA-HC-7750, TCGA-ZG-A9MC, TCGA-XK-AAIR, TCGA-KC-A7F6, TCGA-EJ-5514, TCGA-CH-5762, TCGA-XK-AAK1, TCGA-ZG-A9L9, TCGA-YL-A8SC, TCGA-J4-A83J, TCGA-CH-5792, TCGA-EJ-5518, TCGA-CH-5739, TCGA-CH-5740, TCGA-V1-A9OQ, TCGA-M7-A721, TCGA-EJ-A7NN, TCGA-EJ-5497, TCGA-EJ-A8FO, TCGA-HC-7821, TCGA-XJ-A83G, TCGA-2A-A8VV, TCGA-EJ-A65G, TCGA-V1-A8WS, TCGA-VN-A88M, TCGA-VP-A87D, TCGA-HC-7817, TCGA-ZG-A9L5, TCGA-EJ-7314, TCGA-KK-A7B0, TCGA-G9-6364, TCGA-M7-A725, TCGA-G9-7521, TCGA-ZG-A9LU, TCGA-EJ-8472, TCGA-YL-A9WY, TCGA-EJ-5515, TCGA-V1-A9ZI, TCGA-EJ-7331, TCGA-G9-7519, TCGA-ZG-A9M4, TCGA-EJ-7786, TCGA-VN-A88N, TCGA-G9-6379, TCGA-KC-A4BN, TCGA-V1-A9OH, TCGA-YL-A8SJ, TCGA-ZG-A8QZ, TCGA-HC-A6AO, TCGA-G9-A9S0, TCGA-FC-A6HD, TCGA-G9-A9S7, TCGA-KC-A7FE, TCGA-FC-7961, TCGA-ZG-A8QX, TCGA-HC-A632, TCGA-G9-6342, TCGA-EJ-A8FN, TCGA-HC-A8D1, TCGA-XJ-A9DK, TCGA-KK-A6E7, TCGA-J4-A83K, TCGA-J4-A67T, TCGA-HC-7752, TCGA-VP-A87K, TCGA-YL-A8S9, TCGA-G9-6338, TCGA-G9-6356, TCGA-KK-A7AV, TCGA-HC-A631, TCGA-EJ-7321, TCGA-EJ-5527, TCGA-HC-7212, TCGA-VN-A943, TCGA-VP-A87H, TCGA-EJ-5495, TCGA-TP-A8TT, TCGA-KK-A8IK, TCGA-CH-5772, TCGA-CH-5743, TCGA-YL-A9WJ, TCGA-M7-A71Z, TCGA-2A-AAYU, TCGA-ZG-A9L1, TCGA-KK-A59Z, TCGA-EJ-A65F, TCGA-EJ-5516, TCGA-J4-AATZ, TCGA-VP-A87B, TCGA-HC-8261, TCGA-YL-A8SQ, TCGA-EJ-7325, TCGA-G9-7509, TCGA-J4-A67R, TCGA-HC-8264, TCGA-YL-A8SB, TCGA-EJ-7327, TCGA-CH-5766, TCGA-ZG-A9L2, TCGA-EJ-8470, TCGA-FC-A8O0, TCGA-EJ-7791, TCGA-XJ-A9DX, TCGA-J9-A8CK, TCGA-YL-A8SL, TCGA-G9-6370, TCGA-CH-5763, TCGA-HC-7744, TCGA-HC-7737, TCGA-CH-5765, TCGA-EJ-A65E, TCGA-VP-AA1N, TCGA-G9-6361, TCGA-CH-5789, TCGA-EJ-5496, TCGA-HC-7231, TCGA-HC-A4ZV, TCGA-KK-A6E5, TCGA-YL-A8HK, TCGA-YL-A8S8, TCGA-EJ-5511, TCGA-J4-A67Q, TCGA-HC-7747, TCGA-M7-A720, TCGA-QU-A6IL, TCGA-ZG-A8QW, TCGA-KK-A8IG, TCGA-VN-A88O, TCGA-EJ-5507, TCGA-KK-A8IA, TCGA-HC-A9TE, TCGA-HC-7079, TCGA-KK-A7AY, TCGA-G9-6371, TCGA-M7-A722, TCGA-EJ-7793, TCGA-J4-A83M, TCGA-G9-6343, TCGA-EJ-5517, TCGA-EJ-7797, TCGA-G9-6384, TCGA-EJ-8468, TCGA-J9-A52D, TCGA-V1-A9Z9, TCGA-2A-A8VO, TCGA-ZG-A9LZ, TCGA-SU-A7E7, TCGA-EJ-5519, TCGA-HC-7738, TCGA-XK-AAJP, TCGA-EJ-5522, TCGA-J4-A83I, TCGA-YL-A9WL, TCGA-H9-A6BX, TCGA-CH-5737, TCGA-EJ-7784, TCGA-FC-7708, TCGA-VP-A878, TCGA-G9-7525, TCGA-TP-A8TV, TCGA-YL-A8HJ, TCGA-J4-8198, TCGA-EJ-A46B, TCGA-J4-AATV, TCGA-HC-8260, TCGA-KK-A8IB, TCGA-QU-A6IM, TCGA-KK-A7AQ, TCGA-2A-AAYF, TCGA-XK-AAJR, TCGA-HC-7080, TCGA-KK-A8I4, TCGA-EJ-7792, TCGA-XK-AAJ3, TCGA-M7-A723, TCGA-EJ-5498, TCGA-HC-8216, TCGA-EJ-5506, TCGA-G9-6385, TCGA-G9-6365, TCGA-EJ-A8FP, TCGA-HC-A6AL, TCGA-HC-8266, TCGA-HC-7078, TCGA-EJ-A65J, TCGA-CH-5754, TCGA-EJ-A65M, TCGA-EJ-A7NJ, TCGA-HC-A6HX, TCGA-KK-A8IH, TCGA-HC-7230, TCGA-HC-7081, TCGA-EJ-7782, TCGA-HC-7742, TCGA-EJ-5521, TCGA-EJ-AB20, TCGA-KK-A6E2, TCGA-X4-A8KS, TCGA-Y6-A8TL, TCGA-G9-6373, TCGA-EJ-7317, TCGA-HC-7745, TCGA-KC-A4BR, TCGA-KK-A8I5, TCGA-HC-7213, TCGA-KK-A7B4, TCGA-EJ-A6RC, TCGA-ZG-A9KY, TCGA-G9-A9S4, TCGA-KK-A6E3, TCGA-EJ-A46H, TCGA-EJ-A65D, TCGA-KK-A7AW, TCGA-M7-A724, TCGA-YL-A8SA, TCGA-YL-A8SP, TCGA-EJ-A46I, TCGA-XK-AAJU, TCGA-YL-A9WX, TCGA-J4-A67L, TCGA-J9-A52E, TCGA-HC-7749, TCGA-EJ-5531, TCGA-J4-A67N, TCGA-CH-5790, TCGA-EJ-7312, TCGA-HC-A8D0, TCGA-VN-A88P, TCGA-EJ-A6RA, TCGA-HC-8213, TCGA-V1-A9OY, TCGA-J4-A67S, TCGA-KK-A8IJ, TCGA-EJ-5512, TCGA-CH-5750, TCGA-QU-A6IN, TCGA-J4-A6M7, TCGA-KK-A7AZ, TCGA-CH-5748, TCGA-HC-7077, TCGA-V1-A8WN, TCGA-FC-A4JI, TCGA-KK-A8IM, TCGA-ZG-A9LN, TCGA-G9-6332, TCGA-CH-5794, TCGA-VN-A88K, TCGA-YL-A8SO, TCGA-EJ-5525, TCGA-XQ-A8TB, TCGA-ZG-A9ND, TCGA-2A-AAYO, TCGA-TK-A8OK, TCGA-KK-A8I6, TCGA-ZG-A9LY, TCGA-H9-A6BY, TCGA-ZG-A9LS, TCGA-EJ-5509, TCGA-CH-5769, TCGA-YL-A8HO, TCGA-KK-A59X, TCGA-XK-AAIV, TCGA-VP-A87C, TCGA-KC-A7F5, TCGA-G9-6494, TCGA-HC-A76X, TCGA-J9-A52B, TCGA-VP-A879, TCGA-KK-A7B3, TCGA-XK-AAJA, TCGA-CH-5761, TCGA-X4-A8KQ, TCGA-HC-A6AQ, TCGA-G9-6353, TCGA-KK-A8IC, TCGA-G9-6369, TCGA-EJ-A7NF, TCGA-KK-A7AU, TCGA-V1-A8MF, TCGA-KK-A8ID, TCGA-G9-6351, TCGA-G9-6362, TCGA-KK-A8I9, TCGA-V1-A8WW, TCGA-KK-A6E4, TCGA-EJ-A7NM, TCGA-EJ-7315, TCGA-VP-A872, TCGA-CH-5751, TCGA-YL-A8SK, TCGA-EJ-7794, TCGA-J9-A8CM, TCGA-G9-6366, TCGA-J4-A83L, TCGA-KK-A6E1, TCGA-EJ-A46D, TCGA-VN-A88I, TCGA-CH-5738, TCGA-HC-7819, TCGA-CH-5791, TCGA-KK-A6E8, TCGA-J4-A67M, TCGA-XJ-A83H, TCGA-CH-5753, TCGA-KK-A8IF, TCGA-WW-A8ZI, TCGA-CH-5768, TCGA-G9-6377, TCGA-HC-7740, TCGA-CH-5741, TCGA-EJ-7788, TCGA-HC-8265, TCGA-VP-A876, TCGA-EJ-5499, TCGA-HI-7169, TCGA-QU-A6IP, TCGA-EJ-7330, TCGA-CH-5745, TCGA-KK-A6E0, TCGA-J4-AAU2, TCGA-YL-A8SH | The sample size used here is inconsistent with the differential genes analysis, because some samples lack survival data, so the sample size is reduced. |
