# Supplementary material for: The Landscape of Immune Cells Infiltrating in Prostate Cancer
Source: Front Oncol. 2020 Oct 29;10:517637. doi: 10.3389/fonc.2020.517637 (PMC7658630; doi:10.3389/fonc.2020.517637)
Supplement: Supplementary file 4 [file Table_2.docx]

**Supplementary Table 2：T-test statistical table of 22 immune cells**

**(mean of infiltrating percentage of 22 immune cells ± SD )**

| **Immune cell** | **Normal (n=190)**  **mean%±SD** | **Cancer(n=537)**  **mean%±SD** | ***P*-values** |
| --- | --- | --- | --- |
| Naive B cells | 3.8%±4.7% | 6.5%±8.4% | 0.000 |
| Memory B cells | 2.8%±7.1% | 3.5%±6.1% | 0.272 |
| Plasma cells | 12.4%±8.8% | 12.0%±10.1% | 0.566 |
| CD8+ T cells | 12.8%±9.6% | 9.7%±10.4% | 0.000 |
| Naive CD4+ T cells | 1.0%±3.1% | 1.1%±3.4% | 0.746 |
| Resting CD4+ memory T cells | 11.5%±10.7% | 0.2%±9.8% | 0.167 |
| Activated CD4+ memory T cells | 2.0%±4.9% | 1.7%±3.6% | 0.529 |
| Follicular helper T cells | 3.6%±4.9% | 4.0%±5.3% | 0.358 |
| Regulatory T cells (Tregs) | 3.5%±4.2% | 4.1%±4.6% | 0.090 |
| Gamma delta T cells | 3.3%±5.5% | 2.5%±5.4% | 0.068 |
| Resting NK cells | 0.9%±2.6% | 4.8%±11.7% | 0.000 |
| Activated NK cells | 6.7%±6.8% | 7.0%±7.7% | 0.668 |
| Monocytes | 6.9%±5.8% | 3.9%±5.5% | 0.000 |
| M0 Macrophages | 4.3%±6.0% | 3.5%±6.2% | 0.108 |
| M1 Macrophages | 2.1%±3.0% | 3.1%±4.6% | 0.000 |
| M2 Macrophages | 3.7%±4.3% | 5.4%±5.9% | 0.000 |
| Resting Dendritic cells | 1.7%±3.1% | 3.5%±5.3% | 0.000 |
| Activated Dendritic cells | 2.5%±4.9% | 1.5%±2.3% | 0.014 |
| Resting Mast cells | 11.9%±9.3% | 6.4%±7.7% | 0.000 |
| Activated Mast cells | 1.4%±4.2% | 2.6%±4.9% | 0.001 |
| Eosinophils | 0.7%±2.2% | 1.2%±3.3% | 0.028 |
| Neutrophils | 0.5%±1.9% | 1.6%±3.9% | 0.000 |

**(note: The sum percentage of 22 immune cells was equal to 100%)**
