# Supplementary material for: The Landscape of Immune Cells Infiltrating in Prostate Cancer
Source: Front Oncol. 2020 Oct 29;10:517637. doi: 10.3389/fonc.2020.517637 (PMC7658630; doi:10.3389/fonc.2020.517637)
Supplement: Supplementary file 5 [file Table_3.docx]

**Supplementary Table 3: 193 differential immune genes**

| **Immune gene** | **Normal_Mean** | **Tumor_Mean** | **logFC** | ***p*-value** | **FDR** |
| --- | --- | --- | --- | --- | --- |
| HFE | 3.438184346 | 1.424562906 | -1.271127571 | 1.58E-23 | 8.67E-21 |
| HLA-DMB | 4.84672575 | 15.75918468 | 1.701110541 | 1.20E-05 | 2.98E-05 |
| MR1 | 4.520829667 | 2.038130257 | -1.149341305 | 1.36E-19 | 7.44E-18 |
| ULBP2 | 0.192513917 | 0.562052254 | 1.545741524 | 3.55E-05 | 8.15E-05 |
| PDIA2 | 0.101527234 | 1.89082197 | 4.219074849 | 1.07E-12 | 8.74E-12 |
| CAMP | 2.347852667 | 0.238697434 | -3.298086915 | 1.97E-05 | 4.71E-05 |
| CXCL14 | 9.707854798 | 23.73325553 | 1.289685576 | 6.37E-05 | 0.000140169 |
| SLPI | 492.0103344 | 51.48438138 | -3.25648188 | 7.76E-06 | 1.99E-05 |
| CXCL9 | 3.536138894 | 7.077081461 | 1.000979583 | 0.001048144 | 0.001880868 |
| CXCL11 | 1.998254235 | 4.9250239 | 1.301390579 | 1.50E-06 | 4.28E-06 |
| CXCL6 | 2.617325187 | 1.284899091 | -1.026438119 | 1.34E-09 | 6.27E-09 |
| CXCL13 | 12.34600731 | 3.868133683 | -1.67433499 | 1.41E-08 | 5.54E-08 |
| DEFB1 | 16.5727031 | 7.416623185 | -1.159974556 | 6.81E-12 | 4.75E-11 |
| S100A9 | 26.02470027 | 12.52885145 | -1.054627382 | 0.000254147 | 0.000506083 |
| S100A8 | 4.305278917 | 1.940417379 | -1.149739699 | 0.00014157 | 0.000293711 |
| S100A12 | 0.535769423 | 0.219047066 | -1.290371353 | 3.79E-06 | 1.02E-05 |
| PENK | 4.736673302 | 1.054640829 | -2.167122415 | 1.44E-16 | 2.96E-15 |
| PTGDS | 158.3356977 | 70.55188742 | -1.166229972 | 1.04E-12 | 8.52E-12 |
| TMSB15A | 20.92198377 | 56.20303654 | 1.425628428 | 3.62E-13 | 3.26E-12 |
| S100B | 4.06278306 | 1.640414041 | -1.308408333 | 6.52E-11 | 3.84E-10 |
| S100A6 | 341.7838883 | 155.0596096 | -1.140261452 | 6.34E-16 | 1.08E-14 |
| S100A2 | 11.92970393 | 5.303839423 | -1.169449235 | 2.54E-05 | 5.97E-05 |
| DEFB132 | 5.975454843 | 1.39669435 | -2.097032195 | 4.35E-08 | 1.58E-07 |
| COLEC12 | 6.54322176 | 14.54538114 | 1.152488029 | 2.25E-13 | 2.12E-12 |
| S100A14 | 16.76452905 | 4.096382349 | -2.03298967 | 6.97E-19 | 2.88E-17 |
| S100A16 | 31.83597917 | 14.16150658 | -1.168683382 | 3.57E-16 | 6.59E-15 |
| TINAGL1 | 15.05113548 | 6.711473705 | -1.165170837 | 1.73E-18 | 6.25E-17 |
| WFIKKN1 | 0.200231615 | 0.434566412 | 1.117906887 | 4.99E-09 | 2.11E-08 |
| WFDC2 | 118.5520087 | 27.30728846 | -2.118162137 | 8.70E-19 | 3.43E-17 |
| MMP9 | 2.020203732 | 5.610028522 | 1.473507314 | 2.80E-09 | 1.24E-08 |
| APOBEC3G | 4.252967894 | 1.283057249 | -1.728884421 | 4.01E-19 | 1.82E-17 |
| RBP1 | 7.203402135 | 2.688920463 | -1.421651364 | 2.45E-18 | 8.38E-17 |
| TLR2 | 3.009419138 | 1.296511671 | -1.21484986 | 2.02E-08 | 7.74E-08 |
| PAEP | 52.52792525 | 0.073242689 | -9.486184138 | 0.00174039 | 0.003008518 |
| SFTPA2 | 10.18755918 | 40.5993414 | 1.994647884 | 1.33E-05 | 3.27E-05 |
| RBP4 | 2.619359752 | 1.184279802 | -1.145204241 | 1.45E-07 | 4.85E-07 |
| NOX4 | 0.103089191 | 0.408606226 | 1.986818112 | 3.72E-15 | 5.25E-14 |
| FABP5 | 3.699351717 | 21.12316014 | 2.513481309 | 8.07E-13 | 6.73E-12 |
| CRABP2 | 59.56333227 | 16.65239492 | -1.838694789 | 1.17E-15 | 1.87E-14 |
| CRABP1 | 2.563701667 | 0.691180551 | -1.891093861 | 2.84E-08 | 1.06E-07 |
| DUOX1 | 6.017768296 | 1.524127324 | -1.98124513 | 1.43E-19 | 7.73E-18 |
| NOX1 | 2.038293748 | 0.806295172 | -1.337981992 | 0.000128079 | 0.000267832 |
| PMP2 | 0.661718699 | 0.229609368 | -1.527036542 | 9.73E-12 | 6.58E-11 |
| DUOX2 | 2.538289232 | 0.446935187 | -2.505718933 | 6.48E-16 | 1.11E-14 |
| TLR3 | 2.975337108 | 1.479594862 | -1.00785094 | 1.49E-16 | 3.04E-15 |
| GDF15 | 59.97842744 | 212.7723168 | 1.826794856 | 7.38E-15 | 9.66E-14 |
| SEMG1 | 12218.65533 | 20.52994299 | -9.217140188 | 2.16E-11 | 1.38E-10 |
| CHIT1 | 0.400364188 | 1.614453277 | 2.01166085 | 3.62E-10 | 1.87E-09 |
| CD40 | 9.969515154 | 4.947067477 | -1.010949766 | 3.15E-19 | 1.48E-17 |
| ISG15 | 16.75814598 | 66.30374129 | 1.984227732 | 2.02E-06 | 5.66E-06 |
| MUC4 | 2.292818905 | 0.673762456 | -1.766810465 | 3.91E-05 | 8.91E-05 |
| CST4 | 15.90819595 | 1.650422461 | -3.268862972 | 0.0087099 | 0.013303928 |
| CSRP1 | 196.2238802 | 73.14128386 | -1.423742771 | 5.01E-19 | 2.21E-17 |
| PLA2G2A | 254.3323844 | 868.2223655 | 1.771349413 | 4.15E-05 | 9.41E-05 |
| TPM2 | 352.3428765 | 154.80867 | -1.186493775 | 2.33E-13 | 2.18E-12 |
| FGF2 | 3.271669104 | 1.474688318 | -1.149616775 | 1.20E-13 | 1.19E-12 |
| SEMG2 | 4838.27956 | 9.481028162 | -8.995234897 | 3.85E-06 | 1.03E-05 |
| DES | 1573.496652 | 560.5381054 | -1.489089753 | 2.60E-15 | 3.82E-14 |
| IRF7 | 6.512099096 | 13.77067176 | 1.08040438 | 1.94E-16 | 3.83E-15 |
| ILK | 10.51265635 | 5.20106349 | -1.015248702 | 2.85E-16 | 5.39E-15 |
| APOBEC3C | 34.18275879 | 8.7734956 | -1.962045166 | 1.26E-26 | 1.21E-22 |
| PTGS2 | 51.03731875 | 7.998872259 | -2.673684022 | 1.15E-10 | 6.47E-10 |
| MASP1 | 8.080373638 | 3.029431576 | -1.415374884 | 2.13E-11 | 1.36E-10 |
| PROC | 0.150962962 | 0.904800625 | 2.583405291 | 0.025919771 | 0.036430555 |
| RNASE7 | 0.424544388 | 0.17941696 | -1.242599135 | 1.28E-13 | 1.26E-12 |
| ABCC4 | 29.04463447 | 90.76125364 | 1.643804863 | 1.65E-15 | 2.52E-14 |
| AQP9 | 0.574478783 | 0.249764618 | -1.201684494 | 4.15E-10 | 2.11E-09 |
| BIRC5 | 0.604507953 | 2.277046714 | 1.913330665 | 2.54E-20 | 1.95E-18 |
| ANXA6 | 49.251565 | 22.54568466 | -1.127318246 | 3.05E-18 | 1.00E-16 |
| ALB | 0.134599485 | 2.707799156 | 4.330375937 | 6.53E-09 | 2.71E-08 |
| NOS1 | 0.784699121 | 0.270547924 | -1.536255411 | 9.40E-13 | 7.76E-12 |
| SERPINA3 | 0.431383864 | 1.117523492 | 1.373261043 | 0.007452561 | 0.011537403 |
| CCL18 | 0.735712545 | 2.751668616 | 1.90309264 | 2.92E-10 | 1.53E-09 |
| CCL17 | 0.327268134 | 0.705950042 | 1.109092958 | 0.006958832 | 0.010837973 |
| CCL11 | 0.312056617 | 0.63774523 | 1.0311724 | 4.22E-08 | 1.53E-07 |
| CCL25 | 0.061117747 | 0.229492482 | 1.908783629 | 0.001716553 | 0.002969983 |
| FAM19A1 | 0.467567736 | 0.199932025 | -1.225665799 | 8.63E-11 | 4.96E-10 |
| VAV3 | 2.11519531 | 0.909689194 | -1.217345262 | 1.03E-14 | 1.29E-13 |
| RAC3 | 8.444788731 | 20.92269118 | 1.308935194 | 6.64E-20 | 4.14E-18 |
| CHP2 | 2.882227515 | 0.661308444 | -2.123788995 | 5.97E-17 | 1.37E-15 |
| PRKCB | 3.547398727 | 1.051976583 | -1.753658907 | 4.97E-17 | 1.16E-15 |
| IGHG1 | 35.02986068 | 75.01877674 | 1.098666488 | 0.002177267 | 0.003704764 |
| IGHG2 | 20.14298657 | 64.65767663 | 1.682544062 | 0.001943136 | 0.003332328 |
| IGHV1-69 | 0.549719499 | 1.719977977 | 1.645622533 | 0.008777621 | 0.013402045 |
| IGHV2-5 | 0.478109564 | 1.071659387 | 1.164433266 | 0.009911704 | 0.014970863 |
| IGHV3-15 | 3.996980939 | 9.912690757 | 1.310366031 | 0.012477416 | 0.018520227 |
| IGHV3-23 | 8.993112661 | 21.16526818 | 1.234806321 | 0.022741246 | 0.032267399 |
| IGHV3-49 | 1.945698208 | 4.823658147 | 1.309839712 | 0.008366168 | 0.012829859 |
| IGHV3-66 | 0.432886829 | 1.063070892 | 1.296175996 | 0.032210687 | 0.044488698 |
| IGHV3-7 | 0.168915206 | 0.548626265 | 1.699524487 | 0.00506635 | 0.008088826 |
| IGHV4-31 | 1.546104892 | 3.956994587 | 1.355766893 | 0.004786328 | 0.007674891 |
| IGHV6-1 | 0.154884144 | 0.4490404 | 1.535655788 | 0.006039295 | 0.009507204 |
| IGHV7-81 | 0.13430035 | 0.297379165 | 1.146840506 | 0.005389053 | 0.008569886 |
| IGKV2D-29 | 0.509170469 | 1.84375902 | 1.856429454 | 0.004086628 | 0.006638182 |
| IGLC3 | 15.67755569 | 33.39290245 | 1.090840851 | 0.0114834 | 0.017146617 |
| IGLV2-23 | 7.049832491 | 14.63843784 | 1.054100719 | 0.013915126 | 0.020477016 |
| IGLV3-21 | 5.667461878 | 15.9046667 | 1.488675453 | 0.009138941 | 0.013902935 |
| IGLV6-57 | 2.550926111 | 5.488127102 | 1.105292783 | 0.017863039 | 0.025844905 |
| CXCL17 | 15.93931881 | 5.157897092 | -1.627735081 | 2.64E-06 | 7.26E-06 |
| EDN3 | 2.562894116 | 0.769713063 | -1.73538124 | 1.31E-12 | 1.05E-11 |
| FGF10 | 2.325808998 | 0.603833293 | -1.945510414 | 1.20E-13 | 1.19E-12 |
| SEMA3A | 0.901419284 | 0.29154444 | -1.628482506 | 1.39E-12 | 1.11E-11 |
| SEMA3D | 3.899029462 | 1.824791468 | -1.095383449 | 2.22E-09 | 9.98E-09 |
| SEMA5A | 2.43485744 | 1.15974477 | -1.070029965 | 2.00E-14 | 2.36E-13 |
| SEMA6D | 1.496839606 | 0.484966169 | -1.625963624 | 4.51E-22 | 7.81E-20 |
| SLIT1 | 0.285305128 | 2.214502993 | 2.956405366 | 4.97E-19 | 2.20E-17 |
| CYSLTR2 | 1.776529163 | 0.181373961 | -3.292022118 | 1.10E-07 | 3.74E-07 |
| EDNRA | 18.219499 | 7.75020729 | -1.233176486 | 2.28E-15 | 3.38E-14 |
| EDNRB | 10.39994119 | 3.539343024 | -1.555021874 | 7.15E-11 | 4.17E-10 |
| PLXNA4 | 0.409902749 | 0.188636454 | -1.119673157 | 5.07E-07 | 1.55E-06 |
| ROBO1 | 8.015920348 | 3.646878511 | -1.136206033 | 7.49E-13 | 6.31E-12 |
| ADM2 | 4.069035329 | 9.548889365 | 1.230646136 | 2.04E-15 | 3.07E-14 |
| AMH | 0.096903953 | 0.921182522 | 3.248859617 | 3.23E-13 | 2.94E-12 |
| APLN | 1.339803754 | 3.633873574 | 1.439486529 | 2.20E-09 | 9.93E-09 |
| AREG | 5.503323665 | 1.713066624 | -1.683721918 | 0.034836678 | 0.047775874 |
| BDNF | 0.576031909 | 0.244801529 | -1.234536159 | 7.01E-15 | 9.21E-14 |
| BMP5 | 5.978070289 | 1.231138162 | -2.279687186 | 6.15E-07 | 1.86E-06 |
| CCK | 13.80082748 | 3.592683193 | -1.941621144 | 1.28E-08 | 5.05E-08 |
| CSF3 | 2.039968959 | 0.899206266 | -1.181823206 | 0.000228504 | 0.000458008 |
| CSPG5 | 0.378482063 | 0.806073459 | 1.090686391 | 6.71E-09 | 2.78E-08 |
| CTF1 | 9.742384385 | 4.121521682 | -1.241097821 | 3.42E-22 | 6.26E-20 |
| DKK1 | 7.58347375 | 2.585235205 | -1.55256331 | 3.09E-05 | 7.16E-05 |
| FGF17 | 0.09519835 | 0.219363838 | 1.204317242 | 0.000109477 | 0.000231476 |
| FGF7 | 3.762442046 | 1.35876869 | -1.46936948 | 5.85E-14 | 6.22E-13 |
| GAL | 0.817285781 | 2.780965134 | 1.766673117 | 5.18E-19 | 2.26E-17 |
| GDF10 | 1.103488964 | 0.276429284 | -1.997089839 | 9.19E-12 | 6.24E-11 |
| IAPP | 0.029077256 | 0.278496406 | 3.259695653 | 4.27E-06 | 1.14E-05 |
| IL11 | 0.138986747 | 0.339709545 | 1.289354435 | 1.13E-14 | 1.41E-13 |
| IL1RN | 2.151419608 | 0.850291633 | -1.339259285 | 1.39E-07 | 4.65E-07 |
| IL33 | 13.09151233 | 6.067877868 | -1.109367814 | 3.29E-11 | 2.03E-10 |
| INHA | 0.225592905 | 0.49969215 | 1.147317855 | 4.47E-08 | 1.61E-07 |
| INSL5 | 0.274756491 | 0.709752486 | 1.369162433 | 1.15E-05 | 2.88E-05 |
| KITLG | 8.706118635 | 3.713502145 | -1.229249268 | 6.62E-21 | 6.70E-19 |
| LEFTY1 | 0.489330241 | 0.239511974 | -1.030710662 | 4.30E-09 | 1.83E-08 |
| NDP | 4.164177119 | 1.417915888 | -1.554259481 | 1.24E-16 | 2.58E-15 |
| NPPC | 3.877007028 | 1.904863664 | -1.025255607 | 4.02E-13 | 3.59E-12 |
| NRG1 | 0.51983917 | 0.222101868 | -1.226843821 | 4.56E-17 | 1.07E-15 |
| NRG2 | 1.268195629 | 0.429704268 | -1.561361298 | 8.19E-22 | 1.27E-19 |
| NTF3 | 0.959705871 | 0.44818819 | -1.098487686 | 1.56E-18 | 5.72E-17 |
| NTF4 | 1.795744606 | 0.853326373 | -1.07341264 | 1.10E-13 | 1.10E-12 |
| OGN | 22.30666095 | 8.634067845 | -1.369362241 | 3.39E-08 | 1.25E-07 |
| PDGFC | 8.941835615 | 4.442395968 | -1.009233032 | 8.88E-17 | 1.94E-15 |
| PDGFD | 7.039275577 | 3.209443331 | -1.133103879 | 1.56E-17 | 4.19E-16 |
| PGF | 2.423287519 | 0.887868913 | -1.448546994 | 1.47E-16 | 3.00E-15 |
| PROK1 | 6.002977929 | 2.942435166 | -1.028667736 | 1.38E-08 | 5.45E-08 |
| RABEP2 | 13.16631656 | 26.86881574 | 1.029080945 | 4.60E-17 | 1.08E-15 |
| SCGB3A1 | 24.22262775 | 4.992646777 | -2.278478635 | 1.99E-13 | 1.89E-12 |
| SST | 2.805023281 | 0.911579822 | -1.621571849 | 0.004819817 | 0.007725368 |
| TG | 11.24104769 | 1.825413529 | -2.62248127 | 6.46E-09 | 2.68E-08 |
| TGFB3 | 26.65108479 | 11.49430947 | -1.213274459 | 3.59E-08 | 1.32E-07 |
| THPO | 0.145726672 | 0.4055797 | 1.476720489 | 7.02E-11 | 4.10E-10 |
| TSLP | 2.069677548 | 0.585213793 | -1.822370338 | 4.01E-15 | 5.60E-14 |
| UCN | 0.52870296 | 2.37020164 | 2.164480489 | 6.18E-24 | 4.21E-21 |
| VGF | 0.167865028 | 0.948133201 | 2.497788053 | 1.43E-13 | 1.39E-12 |
| ADCYAP1R1 | 0.517142911 | 0.177270061 | -1.544614118 | 9.98E-13 | 8.22E-12 |
| ADRB1 | 6.318837833 | 12.7407067 | 1.011714157 | 2.16E-13 | 2.04E-12 |
| AGTR1 | 2.562564483 | 6.806085366 | 1.409236938 | 0.018044269 | 0.026082183 |
| ANGPT1 | 6.763595346 | 1.665737441 | -2.021629332 | 5.42E-25 | 8.02E-22 |
| ANGPTL1 | 4.137905363 | 1.091558424 | -1.9225113 | 2.02E-12 | 1.57E-11 |
| ANGPTL3 | 0.059142839 | 0.840627967 | 3.829192055 | 8.17E-14 | 8.39E-13 |
| CNTFR | 4.121835852 | 1.211766685 | -1.766175104 | 4.22E-19 | 1.90E-17 |
| FGFR2 | 11.32814345 | 4.741114468 | -1.256613309 | 1.10E-22 | 3.09E-20 |
| FGFRL1 | 7.432499654 | 22.65935807 | 1.608187595 | 6.48E-23 | 2.19E-20 |
| GHRHR | 0.096055629 | 0.352742892 | 1.876674947 | 3.15E-10 | 1.64E-09 |
| GLP1R | 0.05395841 | 0.349973924 | 2.697327688 | 0.026364703 | 0.036982911 |
| IL18R1 | 1.406990265 | 0.540535067 | -1.380152226 | 3.35E-06 | 9.06E-06 |
| IL1R2 | 0.489120135 | 0.234215423 | -1.062352771 | 8.58E-15 | 1.11E-13 |
| IL1RL1 | 0.741932404 | 0.200212834 | -1.889753292 | 1.91E-07 | 6.27E-07 |
| IL1RL2 | 1.109824102 | 0.510451947 | -1.12048398 | 1.61E-09 | 7.41E-09 |
| IL20RB | 1.017497219 | 0.375375692 | -1.438617716 | 0.001658204 | 0.002876276 |
| IL31RA | 0.1563332 | 0.329430728 | 1.075350935 | 0.019189495 | 0.027571984 |
| LGR6 | 3.902648162 | 0.90096944 | -2.114903327 | 1.24E-19 | 6.90E-18 |
| MET | 5.612491848 | 2.803611638 | -1.001354926 | 2.58E-13 | 2.40E-12 |
| NR4A3 | 7.050465077 | 3.273633076 | -1.1068258 | 1.07E-05 | 2.69E-05 |
| NRP2 | 3.788001915 | 1.773717881 | -1.0946605 | 1.52E-11 | 9.91E-11 |
| PGR | 1.654073037 | 0.610805681 | -1.437237553 | 8.36E-16 | 1.40E-14 |
| PTGER2 | 5.341965973 | 2.640420674 | -1.016602987 | 7.31E-06 | 1.88E-05 |
| PTGER3 | 0.850401488 | 0.411766105 | -1.046319046 | 9.08E-12 | 6.17E-11 |
| SSTR1 | 1.286959753 | 5.338612213 | 2.052497821 | 3.04E-07 | 9.67E-07 |
| SSTR2 | 0.573377744 | 0.241440401 | -1.247818799 | 2.48E-10 | 1.32E-09 |
| TGFBR3 | 10.84004571 | 3.836277769 | -1.498591752 | 6.28E-18 | 1.88E-16 |
| TNFRSF18 | 0.880734025 | 1.936884878 | 1.136959901 | 5.76E-06 | 1.51E-05 |
| TUBB3 | 0.089706671 | 0.232279459 | 1.3725744 | 6.02E-08 | 2.14E-07 |
| SHC2 | 10.38369867 | 21.31513486 | 1.03755776 | 5.41E-16 | 9.44E-15 |
| PRKCA | 6.660025058 | 3.036321758 | -1.133202924 | 1.04E-14 | 1.30E-13 |
| PAK3 | 1.098707946 | 0.332419902 | -1.724729283 | 6.66E-23 | 2.21E-20 |
| CTLA4 | 0.288102812 | 0.645241853 | 1.16325628 | 1.52E-05 | 3.70E-05 |
| CBLC | 10.46806158 | 23.24593821 | 1.150984338 | 4.23E-14 | 4.64E-13 |
| TRGV9 | 2.193279376 | 13.6755208 | 2.640434279 | 8.29E-17 | 1.84E-15 |
| TRGV2 | 1.185663414 | 0.510725925 | -1.215073316 | 0.000824018 | 0.001507238 |
| TRGJP2 | 4.535516175 | 17.46094102 | 1.944792658 | 1.68E-16 | 3.38E-15 |
| TRGC2 | 7.660452625 | 26.16583755 | 1.772182897 | 1.57E-12 | 1.24E-11 |
| TRGC1 | 27.79063867 | 106.7686046 | 1.941816588 | 4.61E-20 | 3.08E-18 |
